# Marek’s Disease Virus (*Gallid alphaherpesvirus* 2)-Encoded miR-M2-5p Simultaneously Promotes Cell Proliferation and Suppresses Apoptosis Through RBM24 and MYOD1-Mediated Signaling Pathways

**DOI:** 10.3389/fmicb.2020.596422

**Published:** 2020-11-03

**Authors:** Zhi-Jian Zhu, Man Teng, Hui-Zhen Li, Lu-Ping Zheng, Jin-Ling Liu, Shu-Jun Chai, Yong-Xiu Yao, Venugopal Nair, Gai-Ping Zhang, Jun Luo

**Affiliations:** ^1^Research Center of Avian Diseases, College of Veterinary Medicine, Sichuan Agricultural University, Chengdu, China; ^2^Key Laboratory of Animal Immunology, Ministry of Agriculture and Henan Provincial Key Laboratory of Animal Immunology, Henan Academy of Agricultural Sciences, Zhengzhou, China; ^3^UK-China Centre of Excellence for Research on Avian Diseases, Henan Academy of Agricultural Sciences, Zhengzhou, China; ^4^College of Animal Science and Veterinary Medicine, Henan Agricultural University, Zhengzhou, China; ^5^The Pirbright Institute and UK-China Centre of Excellence for Research on Avian Diseases, Guildford, United Kingdom; ^6^Jiangsu Co-Innovation Center for the Prevention and Control of Important Animal Infectious Disease and Zoonoses, Yangzhou University, Yangzhou, China; ^7^Key Laboratory of Animal Disease and Public Safety, College of Animal Science and Technology, Henan University of Science and Technology, Luoyang, China

**Keywords:** herpesvirus, *Gallid alphaherpesvirus* 2, oncogenesis, miRNA, miR-M2-5p, RNA-binding protein 24, myogenic differentiation 1, Marek’s disease

## Abstract

MicroRNAs (miRNAs) have been demonstrated for their involvement in virus biology and pathogenesis, including functioning as key determinants of virally-induced cancers. As an important oncogenic α-herpesvirus affecting poultry health, Marek’s disease virus serotype 1 [*Gallid alphaherpesvirus* 2 (GaHV-2)] induces rapid-onset T-cell lymphomatous disease commonly referred to as Marek’s disease (MD), an excellent biological model for the study of virally-induced cancer in the natural hosts. Previously, we have demonstrated that GaHV-2-encoded miRNAs (especially those within the Meq-cluster) have the potential to act as critical regulators of multiple processes such as virus replication, latency, pathogenesis, and/or oncogenesis. In addition to miR-M4-5p (miR-155 homolog) and miR-M3-5p, we have recently found that miR-M2-5p possibly participate in inducing MD lymphomagenesis. Here, we report the identification of two tumor suppressors, the RNA-binding protein 24 (RBM24) and myogenic differentiation 1 (MYOD1), being two biological targets for miR-M2-5p. Our experiments revealed that as a critical miRNA, miR-M2-5p promotes cell proliferation *via* regulating the RBM24-mediated p63 overexpression and MYOD1-mediated IGF2 signaling and suppresses apoptosis by targeting the MYOD1-mediated Caspase-3 signaling pathway. Our data present a new strategy of a single viral miRNA exerting dual role to potentially participate in the virally-induced T-cell lymphomagenesis by simultaneously promoting the cell proliferation and suppressing apoptosis.

## Introduction

MicroRNAs (miRNAs), a class of small non-coding RNAs in length of 22~24 nucleotides, play important roles in regulating gene expression post-transcriptionally in many biological processes, such as cell development, differentiation, immunoregulation, disease progression, and all aspects of cancer biology (Bartel, 2018; Butz and Patocs, 2019; Mei and Zhang, 2019; Nagy et al., 2019). Except for the thousands of cellular miRNAs characterized in metazoan and plants, many miRNAs have also been reported in viruses (miRBase v22^1^). In the last decade, more than 500 miRNAs have been identified in the viral genomes of 34 species, especially in large DNA viruses, such as the human tumorigenic γ-herpesvirus Epstein-Barr virus (EBV), Kaposi’s sarcoma-associated herpesvirus (KSHV), and the avian oncogenic α-herpesvirus *Gallid alphaherpesvirus* 2 (GaHV-2), where some of the viral miRNAs have been demonstrated for their critical roles in virus life cycle, pathogenesis, and/or oncogenesis (Kincaid and Sullivan, 2012; Yao and Nair, 2014; Zhuang et al., 2017; Vojtechova and Tachezy, 2018).

Oncogenic viruses are major pathogens responsible for some of human cancers and most neoplastic diseases of livestock species, such as poultry. The miRNA-mediated regulation of signaling pathways is an important strategy for the oncogenic viruses to induce rapid neoplastic transformation of target cells and development of tumors. For herpesviruses, the most successful virus species to exploit the miRNA pathway either through regulating host miRNAs or through encoding their own miRNAs to modulate host gene expression, miRNA machinery is beneficial to enhance viral pathogenesis, regulate life cycle switch, immune evasion, and promote the establishment of a reservoir of latently infected cells (Cullen, 2013; Grey, 2015; Piedade and Azevedo-Pereira, 2016). For instance, EBV encodes a total of 44 mature miRNAs. Of which, the miRNAs miR-BART6-3p, miR-BART11-5p, miR-BART2-5p, and miR-BHRF1-2-5p regulate both virus and host gene expressions to play important roles in antiviral immunity, lytic reactivation, and development of cancer (Ross et al., 2013; Albanese et al., 2017; Lu et al., 2017; Chen et al., 2019). Among the 25 KSHV-encoded miRNAs, miR-K12-1, miR-K12-3, and miR-K12-11 have been shown to be critical in lymphoproliferation and viral pathogenesis (Happel et al., 2016; Li et al., 2016; Guo et al., 2017; Qin et al., 2017).

*Gallid alphaherpesvirus* 2, commonly known as the Marek’s disease virus type 1 (MDV-1), is an oncogenic α-herpesvirus. Virulent strains of GaHV-2 can establish and maintain latent infections in their natural avian hosts and cause Marek’s disease (MD), a rapid-onset T-cell lymphomatous disease. MD has been regarded as an excellent biomedical model for the study of virally-induced cancers (Osterrieder et al., 2006). GaHV-2 genomes encode 26 miRNAs located in the repeat regions in three gene clusters, namely the Meq-cluster, the mid-cluster, and the LAT-cluster (Burnside et al., 2006; Yao et al., 2008; Luo et al., 2010, 2011). Interestingly, the Meq-cluster miRNAs are expressed at higher levels in lymphomas produced by very virulent plus (vv + MDV) GaHV-2 than those produced by a less virulent (vMDV) GaHV-2 strain, demonstrating a potential significant role of this gene cluster in MD oncogenesis (Morgan et al., 2008). The subsequent studies have confirmed that deletions of the entire Meq-cluster or the single miR-M4 precursor within the cluster from the viruses with different virulence significantly decrease or even abolish virus oncogenicity, implying the critical role of the Meq-cluster in GaHV-2 pathogenesis and/or oncogenesis (Zhao et al., 2011; Yu et al., 2014). The miR-M4-5p, a crucial molecule produced by miR-M4 in the Meq-cluster, has been identified as the functional viral homolog of the cellular miR-155 (Zhao et al., 2009) and regulates both host and viral protein-coding genes (Muylkens et al., 2010; Dang et al., 2017). Similar to miR-K12-11, the other viral miR-155 ortholog encoded by KSHV (Liu et al., 2012), miR-M4-5p has been demonstrated to target TGF-β signaling pathway by downregulating the LTBP1 expression to suppress the maturation and secretion of TGF-β1, as well as to activate the expression of host oncoprotein c-MYC (Chi et al., 2015), triggering the development of MD tumors. The miR-M3-5p in the Meq-cluster was also found to directly target cellular factors involved in antiviral processes including apoptosis, proactively creating a cellular environment beneficial for viral latency and oncogenesis (Xu et al., 2011).

In addition to miR-M4-5p and miR-M3-5p, the other miRNAs in the Meq-cluster may also play critical roles in GaHV-2 oncogenesis. Attractively, miR-M2 is the outstanding one of GaHV-2 miRNA precursors that produces two mature miRNAs, miR-M2-5p and miR-M2-3p, both of which are stably expressed at high levels during virus infection, replication, latency, and even the development of cancers (Yao et al., 2008; Luo et al., 2011; Zhao et al., 2015). Using the bacterial artificial chromosome (BAC) mutagenesis techniques, we had previously generated a series of individual miRNA precursor-deleted mutant viruses and revealed that deletion of miR-M2 from vvMDV genome significantly reduces virus pathogenicity, oncogenicity, and delays the progress of disease (Teng et al., 2015), highlighting its critical role for MD biology. However, the precise molecular regulatory mechanisms mediated by miR-M2 in GaHV-2 oncogenesis remained unclear. Herein, we sought to shed light on the functional roles of miR-M2-5p and elucidate its precise regulatory mechanisms in MD biology. Using hybrid-PCR (Huang et al., 2011), a cDNA library was first produced to screen the candidate host mRNA targets for miR-M2-5p and two host genes *RBM24* and *MYOD1*, known for their important roles in tumor induction, were identified as its biological targets. The data from subsequent experiments suggested the dual role of a single viral miRNA to simultaneously promote the cell proliferation and suppress apoptosis through regulating distinct host target gene-related signaling pathways, which potentially contributes to the virally-induced MD lymphomagenesis.

## Materials and Methods

### Viruses and Cells

Two infectious bacterial chromosome clone (BAC)-derived GaHV-2 viruses, GX0101 and its mutant GXΔmiR-M2 with miR-M2-deletion from the viral genome, were propagated and passaged on confluent chicken embryo fibroblast (CEF) monolayers as previously described (Teng et al., 2015). Primary CEF cultures were prepared from 9-day-old embryonated eggs (Jinan SPF Egg & Poultry Co., China) and were maintained in medium 199 (Gibco, United States) containing 5% fetal bovine serum (FBS; Hyclone, United States). Chicken lymphoblastoid (MDCC-MSB-1) cells (gift from Prof. Ai-Jian Qin, Yangzhou University, China) were maintained in RPMI 1640 medium (Gibco, United States) supplemented with 10% FBS (HyClone, United States) and 0.5% penicillin/streptomycin solution (Sigma-Aldrich, United States). Human embryonic kidney cells 293T (HEK293T; American Type Culture Collection, VA, United States) were maintained in Dulbecco’s modified Eagle’s medium (DMEM; Invitrogen, United States) supplemented with 10% FBS, 0.5% penicillin and streptomycin (Sigma-Aldrich, United States). All of the cell lines were incubated at 37°C in a humidified chamber with 5% CO_2_.

### Antibodies and Reagents

The primary antibodies against the host proteins RBM24 (ab94567; Abcam, United States), Bcl-2 (610538; BD Biosciences, United States), IGF2 (MAB2921; R&D, United States), Caspase-3 (#9662S), and p63 (#13109S; Cell Signaling Technology, United States), together with those anti β-actin (sc-47778), MYOD1 (sc-377460), Bcl-xL (sc-8392), and Pax3 (sc-376215) from Santa Cruz Biotechnology (United States), were used in the present study. The specificity of the antibodies against avian host have been verified in the study. The goat anti-mouse and goat anti-rabbit horseradish-peroxidase (HRP)-conjugated secondary antibodies were purchased from Amersham Pharmacia Biotech (United States). Cisplatin, as intrinsic apoptotic stimulus, was obtained from Beyotime (S1552). The synthetic siRNAs, miR-M2-5p mimics and negative controls listed in Supplementary Table S1 were provided by GenePharma (Shanghai, China).

### Primer Design for Hybrid-PCR

Based on the sequence of miR-M2-5p, a specific reversal and complementary hybrid-primer was designed to recognize the putative miRNA binding sites located in the candidate host mRNA targets. Since G:U base pairs are allowed for the miRNA/mRNA duplexes, the miR-M2-5p hybrid-primer were synthesized as a compatible primer: Adenines (A) located in miRNA hybrid-primer was substituted by random insertions of adenines (A) or guanines (G). All primers used for hybrid-PCR are summarized in Supplementary Table S2.

### The Hybrid-PCR and Sequencing

The first-strand cDNA was synthesized using the total cellular RNA extracted from CEF cells as templates and then the hybrid-PCR was performed with two rounds of PCR reactions as described previously (Huang et al., 2011). The first and second rounds were carried out with the miR-M2-5p hybrid-primer and the 3'-RACE out or inner primer provided by the 3'-Full RACE Core Set (TaKaRa), respectively. All PCR products were gel-purified, cloned into pMD19-T vector (TaKaRa) and then transformed into *Escherichia coli* DH5α competent cells to produce a cDNA library containing the partial sequences of mRNAs potentially targeted by miR-M2-5p. The positive clones randomly selected and identified by PCR analysis were sent to Sangon Biotech Co., Ltd. (Shanghai, China) for DNA sequencing. All sequences obtained were further analyzed using the online bio-software BLASTn^2^ to identify the host genes as the primary candidate targets for miR-M2-5p.

### Bioinformatics Prediction

All genes having perfect base pairing to the seed region of miR-M2-5p obtained from the hybrid-PCR assay were used for further prediction of mRNA targets, utilizing the bio-software “RNAhybrid” (Rehmsmeier et al., 2004). For target screening, the criteria are set as follows: a perfect Watson Crick match between mRNA and miRNA at position 2–7 and allowance of G:U pairs in the seed sequence.

### Plasmid Construction

In order to characterize the candidate targets, the 3'UTRs of primarily identified host mRNAs containing the miR-M2-5p binding sites were amplified by PCR using the corresponding primer pairs (Supplementary Table S3) and then cloned into psiCHECK-2 vector *via Not* I and *Xho* I sites (Promega) to construct the dual-luciferase reporter plasmids. The mutant 3'UTR with changed binding sites were prepared by annealing the oligonucleotides (Supplementary Table S3) and cloned into psiCHECK-2 vector in the same way. All primers and oligonucleotides were synthesized by Sangon Biotech Co., Ltd. (Shanghai, China).

### Dual Luciferase Reporter Assay

The miRNA targets verification assay were performed in HEK293T cells. The wild type or mutant 3'UTR dual-luciferase reporter (200 ng each) along with the miR-M2-5p or negative control (50 nM each) were co-transfected into HEK293T cells using Lipofectamine 2000 reagent (Invitrogen) in 48-well plates. At 48 h post-transfection (hpt), cells were washed twice with phosphate buffered saline (PBS, pH 7.4) and lysed with 20 μl 1 × passive lysis buffer (PLB) for 15 min at room temperature. The activities of Firefly and Renilla luciferase were measured using the Dual-Luciferase® Reporter Assay system (Promega, United States). All data were acquired from three independent repeats and calculated as means (M) ± standard deviations (SD) utilizing the software “GraphPad Prism” (version 6.0).

### Transient Transfection

To investigate whether miR-M2-5p downregulates the gene expression of the candidate targets *in vitro*, CEF monolayers in six-well plates were transfected with 50 nM of miRNA duplex mixed with 6 μl X-tremeGENE HD DNA transfection reagent (Roche, Switzerland) in 300 μl Opti-MEM (Invitrogen, United States). At 24 and 48 hpt, the cells were collected for reverse transcription quantitative PCR (RT-qPCR) and western blot analysis. To further confirm the involvement of the identified mRNA targets, specific small interfering RNAs (siRNAs) listed in Supplementary Table S1 were used for silencing the expression of corresponding genes. Transient transfections, RT-qPCR, and western blot analysis were performed as described above. For miRNA interference test, 1 × 10^6^ of MSB-1 cells were resuspended in 100 μl Opti-MEM medium and mixed with 50 nM of miRNA inhibitor duplex or NC (Supplementary Table S1). Then the electroporation was performed using NEPA21 Electroporator (Nepa Gene Co., Ltd., Japan) with optimized condition at voltage 175 V and a pulse width 1 ms of poring pulse. At 24 h post electroporation, cells were harvested for RT-qPCR and western blot analysis.

### Virus Infection

The confluent CEF monolayers in six-well plates were infected with GaHV-2 viruses GX0101 or GXΔmiR-M2 (100 PFU/well) and maintained at 37°C in a 5% CO_2_ incubator. The same amount of uninfected CEF cells equivalent to the infected CEF cell number in the infected group was added in the mock groups and was maintained in the same conditions. At 72, 96, and 120 h post-infection (hpi), cell cultures were collected for further experiments.

### Reverse Transcription and Quantitative Real-Time PCR

The total RNA was extracted from CEF or MSB-1 cells using the TRIzol reagent (Invitrogen). Reverse transcription of mRNA was performed with PrimeScript™ RT Master Mix (Perfect Real-Time; TaKaRa) according to the manufacturer’s instructions. Reverse transcription (RT) of miRNA was performed with stem-loop structured reverse primers (Supplementary Table S4) using PrimeScript™ RT Reagent Kit (TaKaRa). With TB Green® Premix Ex Taq™ II (TaKaRa), RT-qPCR amplification was carried out in 7500 Fast Real-Time PCR Systems (Applied Biosystems, Life Technologies, United States) according to the manufacturer’s protocols. *GAPDH* and *U6* gene expressions were also detected as the internal controls of mRNA or miRNA, respectively. All primers used for RT-qPCR analysis are listed in Supplementary Table S4. Relative quantification of the target gene expression was calculated with 2^−ΔΔct^ method. For the absolute quantification of miRNA expression, synthetic miR-M2-5p and mut-miR-M2-5p mimics (GenePharma Shanghai, China) were used as the standards.

### Measurement of Cell Viability

A commercial cell counting Kit-8 (CCK-8; Beyotime, China) was used to assess the influence of miR-M2-5p expression on CEF proliferation activity. Briefly, CCK-8 (10 μl per well) was added onto CEFs in 96-well plates infected with GaHV-2 viruses, over-expressing miR-M2-5p or specific siRNAs and the plates were incubated at 37°C for 1 h, and then the absorbance at 450 nm was measured using a iMark™ Microplate Reader (BIO-RAD). For the MSB-1 cells transfected with miR-M2-5p inhibitor, the CCK-8 assay was performed using the same method. Each group of the experiments was independently repeated in triplicates and the data were calculated as M ± SD.

### Apoptosis Analysis

Apoptosis was evaluated by annexin V-fluorescein isothiocyanate/propidium iodide (FITC/PI) assay. At different time points, the CEF cells over-expressing miRNAs, siRNAs or infected with GaHV-2 viruses in six-well plates were treated with 5 μM cisplatin, an apoptosis inducer to increase apoptosis rate, and incubated at 37°C for 4 h. Then the cells were collected by trypsinization without ethylenediaminetetraacetic acid (EDTA). The cells were washed twice with PBS and stained using the annexin V-FITC/PI apoptosis detection kit (Beyotime, Shanghai, China) according to the manufacturer’s instructions. CytoFLEX flow cytometry was performed to analyze cell apoptosis levels. The MSB-1 cells transfected with miR-M2-5p inhibitor were directly collected without trypsinization, and the apoptosis level of cells was evaluated as described above. Each group of the experiments was independently repeated in triplicates and the cell viability was determined by an Alamar blue assay (AbD Serotec, Oxford, United Kingdom).

### Western Blot Analysis

The treated or mock CEF or MSB-1 cells mentioned above were lysed for whole-cell extracts with RIPA buffer (Solarbio, Beijing, China) supplemented with protease inhibitors (Roche, United States). Protein concentration of the fractions was determined using the Micro BCA Protein Assay Reagent kit (Thermo Fisher Scientific). The cell lysate with equal volume of SDS-PAGE loading buffer were run on 10% SDS-PAGE gels and transferred to PVDF membranes. Membranes were blocked in 5% nonfat milk and incubated with the corresponding protein-specific primary antibodies overnight at 4°C, followed by the incubation with HRP-conjugated secondary antibodies for 2 h at room temperature. Subsequently, Luminescent signals were detected with western blotting chemiluminescence reagent (Perkin Elmer, United States) and recorded by Vilber Lourmat (Vilber, Paris, France). Where indicated, band intensities were quantified using Evolution-Capt software and shown in the figures as relative band intensity normalized to β-actin.

### Statistical Analysis

Unpaired two-tailed Student *t*-tests were conducted using SPSS software to determine the statistical significance of the data obtained from different experimental groups. All graphs represent the M ± SD of normalized data for triplicate samples from each of three independent repeats. Values of *p* were used for statistical analyses.

## Results

### Putative Host mRNA Targets for miR-M2-5p Acquired by Hybrid-PCR

**Figure 1:**
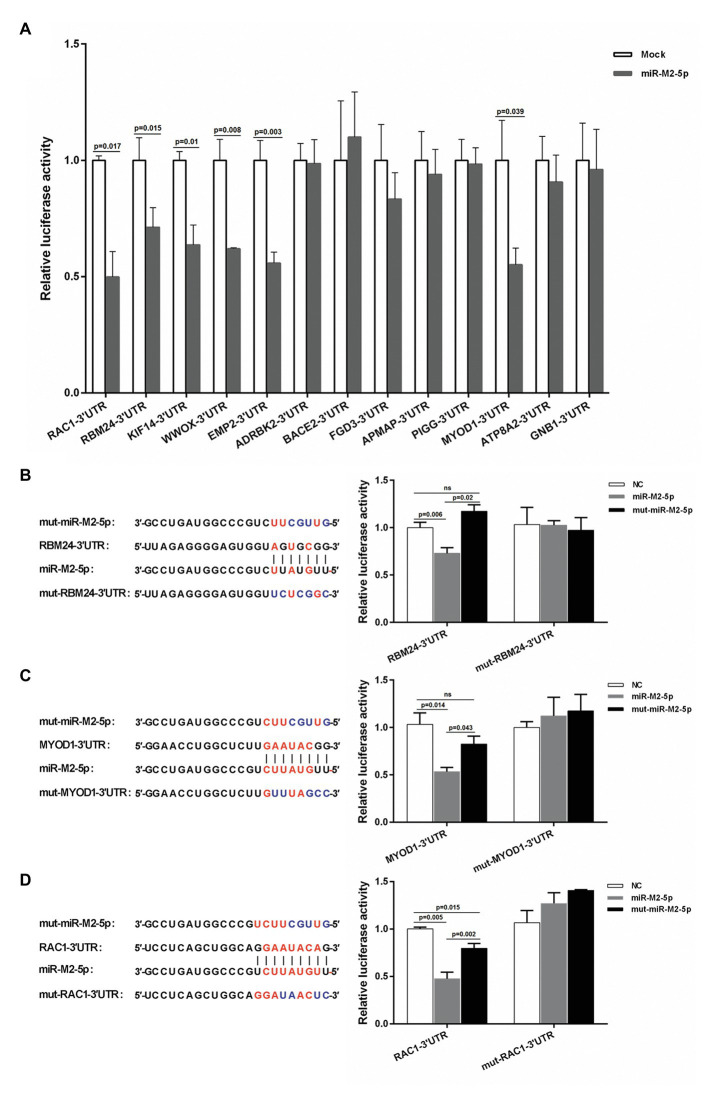
Interactions between miR-M2-5p and 3'UTRs of candidate mRNA targets determined by dual luciferase reporter assay (DLRA). **(A)** The first round of DLRA for primary analysis of the interactions between miR-M2-5p and 3'UTRs of 13 mRNA containing perfect base pairing to the seed region of miR-M2-5p, including *RBM24*, *MYOD1*, *RAC1*, *ADRBK2*, *WWOX*, *EMP2*, *KIF14*, *BACE2*, *FGD3*, *APMAP*, *PIGG*, *ATP8A2*, and *GNB1*. **(B–D)** DLRA performed for confirming the interactions between miR-M2-5p and 3'UTRs of six distinct mRNA candidate targets, *RBM24*, *MYOD1*, and *RAC1*. The colored seed sequence of miRNA or binding sites of 3'UTRs and the corresponding mutants are shown on the left-hand side. The wild type or mutated 3'UTRs of candidate mRNA targets were cloned into downstream of *Renilla* luciferase in psiCHECK-2 vector, and then were co-transfected into 293T cells with the miR-M2-5p, mut-miR-M2-5p, or miRNA negative control (NC) mimics, respectively. *Firefly* and *Renilla* luciferase activities were measured at 48 h post-transfection (hpt) using the dual luciferase reporter system (Promega). *Firefly* luciferase was served as the internal control. For each luciferase assay, relative luciferase activity was normalized with respect to miRNA NC. Results are shown as the mean ± SD of three independent experiments. Independent sample *t*-test was used to analyze the statistical differences between groups. Values of *p* indicated on columns were used for statistical analyses; ns, no significance.

To reveal the regulatory mechanisms mediated by GaHV-2-encoded miR-M2-5p in MD oncogenesis, a hybrid-PCR was first performed in triplicate to construct a cDNA library for screening the putative host mRNA targets. A total of 400 clones from each trial were randomly selected for PCR analysis and about 600 positive ones were sequenced for identification of the unique candidate genes. Finally from the cDNA library, as listed in Supplementary Table S5, a total of 51 putative targets for miR-M2-5p were primarily obtained. Among these, there were 25 candidate mRNA genes containing the miRNA binding sites in 3'UTRs and 13 of them had perfect base pairing to the seed region of miR-M2-5p, which were further analyzed and predicted as candidate targets utilizing bio-software “RNAhybrid” (Rehmsmeier et al., 2004). The thirteen candidates, including *RBM24*, *MYOD1*, *RAC1*, *ADRBK2*, *WWOX*, *EMP2*, *KIF14*, *BACE2*, *FGD3*, *APMAP*, *PIGG*, *ATP8A2*, and *GNB1*, were chosen as the priority target candidates of miR-M2-5p for further investigation. The site-directed dual luciferase reporter assays (DLRA) were performed to determine *in vitro* interactions between the miR-M2-5p and 13 3'UTRs containing potential target sites. For the first round of DLRA analysis, the relative luciferase activities of six 3'UTR reporters, including *RAC1*, *RBM24*, *KIF14*, *WWOX*, *EMP2*, and *MYOD1*, were observed to be significantly repressed by miR-M2-5p, compared to the negative control (NC) mimics (*p* < 0.05; Figure 1A). To ensure that the downregulation of target reporter genes by miR-M2-5p is seed sequence dependent, the mutated miR-M2-5p mimics were used for the second round of DLRA analysis. The results demonstrated that once the seed sequence of miR-M2-5p was mutated, its repression functions to the target reporters were all significantly lost (Figures 1B–D and Supplementary Figures 1A–C). To further confirm that the repressions by miR-M2-5p on the 3'UTR reporters are specifically dependent on the predicted binding sites, we generated six corresponding 3'UTR reporter mutants, named as mut-RBM24-3'UTR, mut-MYOD1-3'UTR, mut-RAC1-3'UTR, mut-WWOX-3'UTR, mut-EMP2-3'UTR, and mut-KIF14-3'UTR. As expected, the DLRA analysis showed that all of the mutated luciferase reporters lost their responses to miR-M2-5p (Figures 1B–D and Supplementary Figures 1A–C).

### miR-M2-5p Overexpression Downregulates the Expression of Candidate Targets

**Figure 2:**
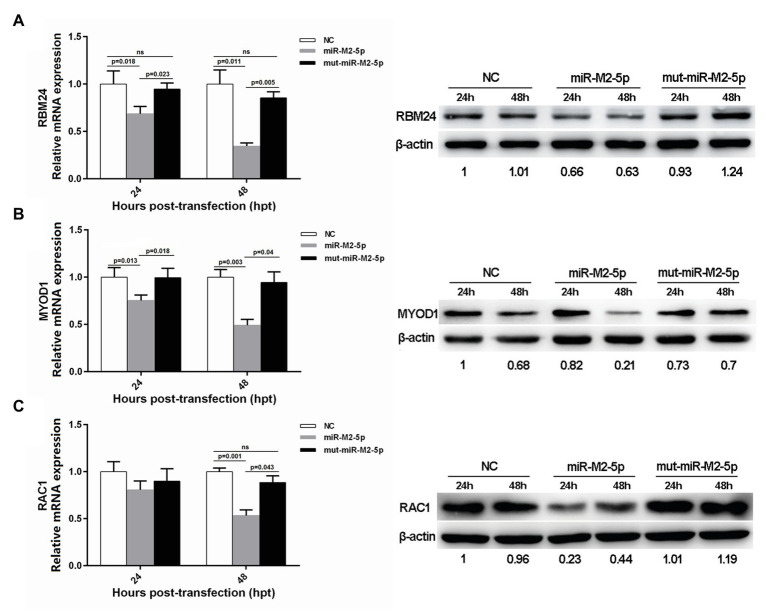
Expression levels of three candidate host targets in chicken embryo fibroblast (CEFs) overexpressing miR-M2-5p determined by reverse transcription quantitative PCR (RT-qPCR; left panels) and western blot (WB) analysis (right panels). **(A)** miR-M2-5p overexpression significantly inhibits RNA-binding protein 24 (RBM24) mRNA and protein expressions in CEFs. **(B)** miR-M2-5p overexpression significantly inhibits myogenic differentiation 1 (MYOD1) mRNA and protein expressions in CEFs. **(C)** miR-M2-5p overexpression significantly inhibits RAC1 mRNA and protein expressions in CEFs. Cells were lysed at the indicated times and subjected to immunoblot analysis for the indicated proteins. Numbers below the blots indicate relative band intensity normalized to β-actin. Error bars are derived from three independent replicates. Values of *p* indicated on columns were used for statistical analyses; ns, no significance.

In order to determine whether the miR-M2-5p recognizes candidate mRNA targets, expression levels of the six candidate targets were evaluated in CEFs overexpressing miR-M2-5p, mut-miR-M2-5p, or NC mimics. As demonstrated on the left panels in Figure 2, the RT-qPCR analysis showed that miR-M2-5p overexpression significantly reduced the mRNA expression levels of host genes *RBM24*, *MYOD1*, and *RAC1* at 24 and/or 48 hpt. The western blot analysis further confirmed the downregulation of *RBM24*, *MYOD1*, and *RAC1* expression as a consequence of miR-M2-5p overexpression (Figure 2, right panels). However, the expression of the other three genes, *WWOX*, *EMP2*, and *KIF14*, were not significantly repressed by miR-M2-5p, compared to the mut-miR-M2-5p and NC mimics either at the transcript or the protein levels (Supplementary Figure 2). These results suggested that the host genes *RBM24*, *MYOD1*, and *RAC1* are most likely the genuine direct targets of miR-M2-5p.

### Expressions of Host RBM24 and MYOD1 Genes Are Downregulated by Virus-Expressed miR-M2-5p

**Figure 3:**
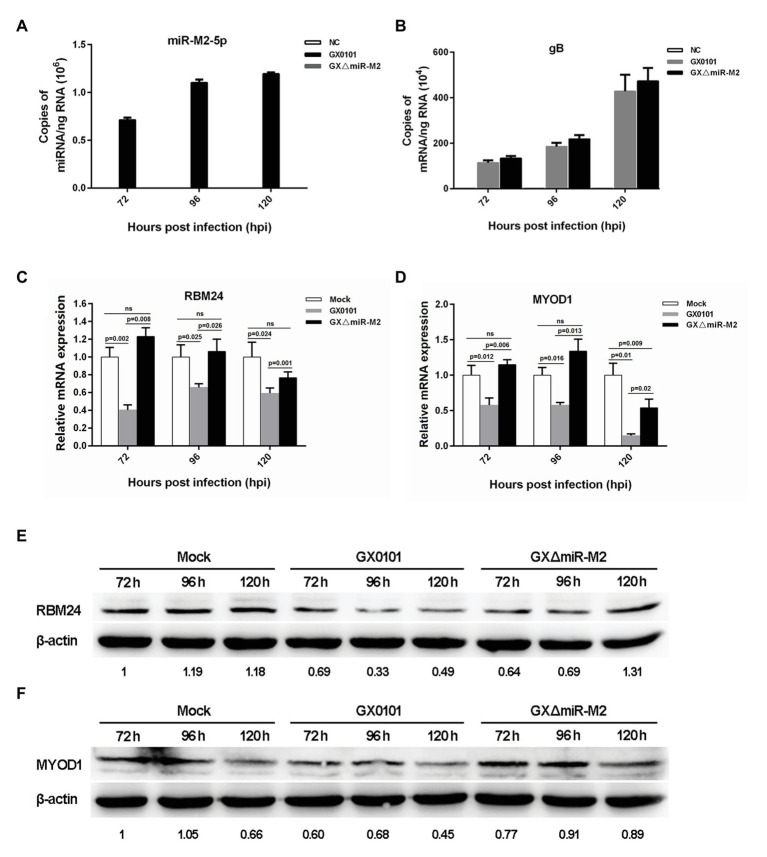
Expression levels of candidate host targets in CEFs infected with *Gallid alphaherpesvirus* 2 (GaHV-2). **(A,B)** Expression levels of gB and miR-M2-5p in virus-infected CEFs determined by RT-qPCR. **(C,D)** Expression levels of the *RBM24* and *MYOD1* mRNA genes in virus-infected CEFs determined by RT-qPCR. Results are shown as the mean ± SD of three independent experiments. Independent sample *t*-test was used to analyze the statistical differences between groups. Values of *p* indicated on columns were used for statistical analyses; ns, no significance. **(E,F)** Expression levels of the RBM24 and MYOD1 proteins in virus-infected CEFs determined by western blot. Numbers below the blots indicate relative band intensity normalized to β-actin.

In order to further verify whether the three candidate targets *RBM24*, *MYOD1*, and *RAC1* are indeed targeted by miR-M2-5p, the intracellular miRNA/mRNA interactions were investigated utilizing the GaHV-2 infection system with strains GX0101 and its mutant GXΔmiR-M2, in which the miR-M2 precursor had been deleted from the viral genome. The virus-infected cell cultures were collected at different time points for RT-qPCR analysis to confirm the expression of miR-M2-5p in the wild type virus and its successful deletion in mutant virus GXΔmiR-M2 (Figure 3A). The expression of MDV gB gene were determined to confirm the successful infection and propagation of viruses in CEFs (Figure 3B). The expression levels of *RBM24* and *MYOD1*, as demonstrated in Figures 3C,D, were significantly downregulated in GX0101-infected cells, compared to that of the mock or GXΔmiR-M2-infected groups. The western blot analysis of the two host genes in virus-infected cells were consistent with RT-qPCR analysis further confirming the observation that the *RBM24* and *MYOD1* are being the biological targets for miR-M2-5p (Figures 3E,F). However, no significant difference of *RAC1* expression levels among the different experimental groups was observed (Supplementary Figures 3A,B). Interestingly, expression of both *RBM24* and *MYOD1* was not fully recovered to mock levels in miRNA mutant virus-infected CEF cells, possibly caused by the interference of the other viral miRNA or protein components participated in GaHV-2 infection.

### miR-M2-5p Enhances Cell Viability and Inhibits Cell Apoptosis

**Figure 4:**
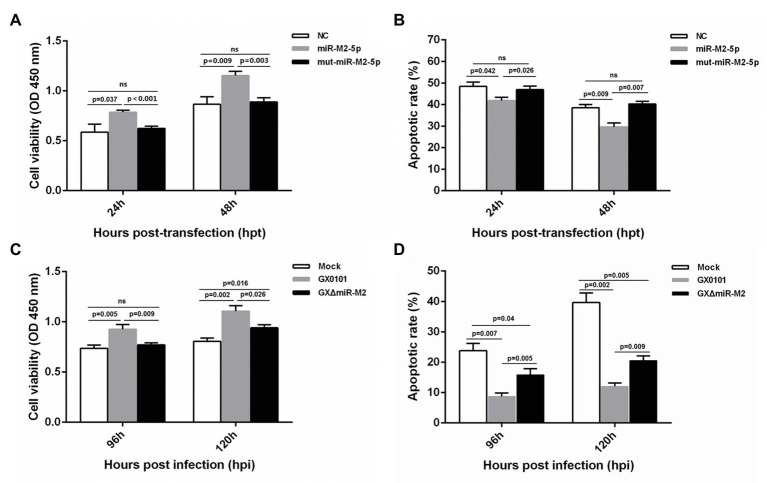
Cell viability and apoptosis of miR-M2-5p-overexpressed **(A,B)** or GaHV-2-infected **(C,D)** CEF cells. At different time points post miRNA transfection or virus infection, the CEF cells were harvested for Annexin V-FITC and propidium iodide (PI) staining for apoptosis analysis using flow cytometry. CCK-8 assay was used to assess cell viability. Hpt, hours post-transfection; Hpi, hours post-infection. CEFs without transfection or infection serve as mock controls. Data are shown as mean ± SD of three independent experiments. Independent sample *t*-test was used to analyze the statistical differences between groups. Values of *p* indicated on columns were used for statistical analyses; ns, no significance.

For further exploration of miR-M2-5p function, we performed assays to identify the effects of miR-M2-5p on both cell viability and cell apoptosis. Compared to the mut-miR-M2-5p or NC mimics-transfected cells, CCK-8 assay showed that the cell viability of CEFs was significantly increased post-transfection with miR-M2-5p (Figure 4A). Next, the apoptosis-blocking capability of miR-M2-5p was determined by annexin V-FITC/PI staining, and the results showed that miR-M2-5p remarkably reduced the cisplatin-induced apoptosis in CEF cells (Figure 4B). Especially at 48 hpt, the overexpression of miR-M2-5p induced significant decrease of the cisplatin-induced apoptosis from 38.52 to 29.6% compared to NC mimics, and from 40.26 to 29.6% compared to mut-miR-M2-5p.

Enhanced cell viability was further observed in GaHV-2-infected cells, which are normally expressing the virus-encoded miR-M2-5p (Figure 4C). Likewise, CEF cells infected with GaHV-2 viruses were analyzed for the resistance to cisplatin-induced apoptosis. As shown in Figure 4D, cells infected with GX0101 manifested a significant decrease in cisplatin-induced apoptosis. However, compared to the parental GX0101 virus, deletion of miR-M2-5p from the viral genome (GXΔmiR-M2 group) significantly rescued the inhibition of GaHV-2 on cell apoptosis although it is not completely back to mock levels (Figure 4D). We consider it as a function of inhibiting apoptosis that possibly induced by the other viral components (Osterrieder et al., 2006; Xu et al., 2011). Taken together, the data obtained from both of the miRNA overexpression and virus-infected CEFs indicated that miR-M2-5p promotes cell viability and inhibits cell apoptosis.

### miR-M2-5p Targets RBM24 to Activate p63 Expression and Promote Cell Proliferation

**Figure 5:**
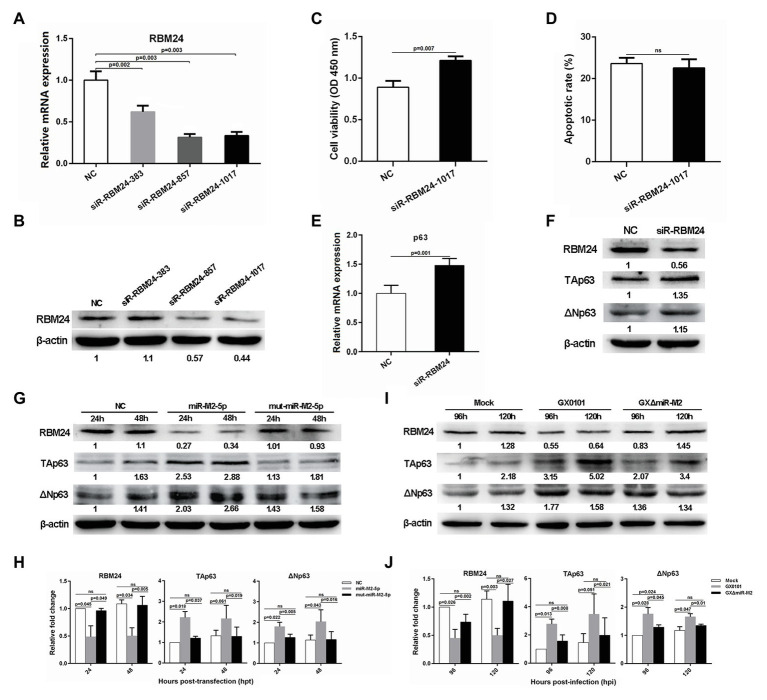
The RBM24-mediated p63 gene expressions and cell viability. **(A,B)** The mRNA and protein expression levels of RBM24 in CEFs overexpressing distinct siRNAs. **(C,D)** Cell viability and apoptosis of the RBM24-silenced CEF cells. CEF cells transfected with RBM24 siRNA were harvested at 48 hpt for Annexin V-FITC and propidium iodide (PI) staining for apoptosis analysis using flow cytometry. CCK-8 assay was used to assess cell viability. Data are shown as the mean ± SD of three independent experiments. **(E,F)** RT-qPCR and western blot analysis of p63 expressions in the RBM24-silenced CEF cells at 48 hpt. **(G)** Expressions of RBM24 and p63 proteins in CEFs overexpressing miR-M2-5p. **(H)** Relative signal intensities in western blot analysis of the RBM24 and p63 proteins in CEFs overexpressing miR-M2-5p. **(I)** Expressions of RBM24 and p63 proteins in GaHV-2-infected CEFs. **(J)** Relative signal intensities in western blot analysis of the RBM24 and p63 proteins in GaHV-2-infected CEFs. Numbers were normalized to the corresponding signal from the β-actin bands. Error bars are derived from three independent replicates. Values of *p* indicated on columns were used for statistical analyses; ns, no significance.

Considering that RBM24 is an important post-transcriptional regulatory factor participating in the regulation of mRNA stabilization and translation (Kiledjian et al., 1995; Krecic and Swanson, 1999; Poon et al., 2012), we synthesized three specific siRNAs to silence RBM24 expression to validate its potential role in the miR-M2-5p promoted cell growth. For the three *RBM24*-specific siRNAs transfected CEF cells, siR-RBM24-1017 demonstrated the best repression effect on both of the mRNA and protein expression levels and was used for further experiments (Figures 5A,B). In addition, CCK-8 assay showed that silencing of RBM24 induced a significantly increased cell viability (Figure 5C). However, no significant difference was observed in the levels of cell apoptosis between the siRNA-transfected and negative control groups (Figure 5D).

Previous studies have reported that RBM24 is involved in the regulation of *p63* and *p21* mRNA stability by binding to the AU-rich elements (AREs) in their 3'UTRs in different human cancer cell lines (Jiang et al., 2014; Xu et al., 2014). Sequence comparison between the chicken and mammalian RBM24 proteins showed that they share a high degree of similarity (data not shown). However, sequence analysis of chicken genes *p63* and *p21* shows rich AREs in the former rather than in the latter. We performed RT-qPCR analysis to determine the mRNA expression levels of *p63* or *p21* and found that compared to the mock transfected cells, *p63* expression level was significantly upregulated in *RBM24*-silenced cells (Figure 5E), while the expression of *p21* was not significantly affected (Supplementary Figure 4). Furthermore, western blot analysis showed that in RBM24-silenced cells, the expressions of two p63 protein isoforms, TAp63 and ΔNp63, are consistent with that of their mRNA expressions (Figure 5F). In order to verify that the change in *p63* expression was caused by miR-M2-5p, we assessed the p63 expression level in CEF cells overexpressing miR-M2-5p. Compared to the mut-miR-M2-5p or NC mimics, miR-M2-5p increased p63 expression, as shown in Figures 5G,H, accompanying with the downregulation of RBM24 protein. Meanwhile, compared to the mock and GXΔmiR-M2 group, CEFs infected with parental GX0101 virus showed also higher levels of p63 (Figures 5I,J). These data indicate that miR-M2-5p-induced downregulation of RBM24 activates p63 expression and promotes the proliferation of CEF cells.

### miR-M2-5p Regulates the MYOD1-Mediated Signaling Pathways to Promote Cell Proliferation and Inhibit Cell Apoptosis

**Figure 6:**
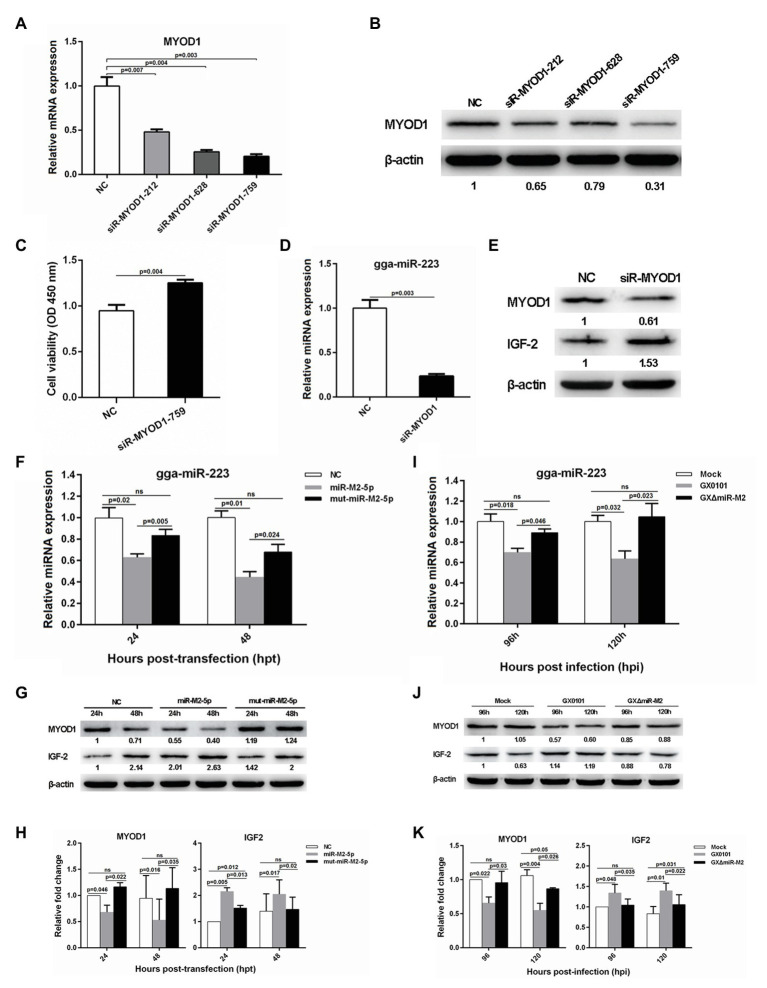
The MYOD1-mediated IGF2 gene expressions and cell viability. **(A,B)** mRNA and protein expression levels of MYOD1 in CEFs overexpressing distinct siRNAs. **(C)** Proliferation analysis of MYOD1-silenced CEF cells. CEF cells transfected with MYOD1 siRNAs were harvested at 48 hpt for CCK-8 assay. **(D,E)** RT-qPCR and western blot analysis of gga-miR-223 and IGF2 expressions in MYOD1-silenced CEF cells at 48 hpt. **(F,G)** Expression levels of gga-miR-223, MYOD1, and IGF2 proteins in CEFs overexpressing miR-M2-5p determined by RT-qPCR or western blot analysis. **(H)** Relative signal intensities in western blot analysis of the MYOD1 and IGF2 proteins in CEFs overexpressing miR-M2-5p. **(I,J)** Expression levels of gga-miR-223, MYOD1, and IGF2 proteins in GaHV-2-infected CEFs determined by RT-qPCR or western blot analysis. **(K)** Relative signal intensities in western blot analysis of the MYOD1 and IGF2 proteins in GaHV-2-infected CEFs. Numbers were normalized to the corresponding signal from the β-actin bands. Error bars are derived from three independent replicates. Values of *p* indicated on columns were used for statistical analyses; ns, no significance.

To investigate the potential role of *MYOD1* targeting by miR-M2-5p in GaHV-2 oncogenesis, we efficiently knocked down the expression of MYOD1 using the siR-MYOD1-759 (Figures 6A,B), and found that the silence of MYOD1 substantially increase the cell viability (Figure 6C). Early study has shown that MYOD1 can upregulate miRNA-223 to suppress IGF2 expression and inhibit cell proliferation (Li et al., 2017). Thus we have analyzed the gga-miR-223-mediated IGF2 signaling in MYOD1-silenced cells and confirmed that the siRNA-induced gene silence of MYOD1 significantly decreased gga-miR-223 expression and upregulated the IGF2 expression (Figures 6D,E). To confirm that the MYOD1-mediated IGF2 signaling is regulated by miR-M2-5p, we further analyzed the expressions of gga-miR-223 and IGF2 in CEF cells overexpressing miR-M2-5p. As expected, miR-M2-5p decreased the transcription level of gga-miR-223 (Figure 6F) and increased the IGF2 expression, accompanying with the downregulation of MYOD1 expression (Figures 6G,H). In the further virus infection assays, GaHV-2-encoded miR-M2-5p also showed similar down or up regulations of the expressions of gga-miR-223 and IGF2 (Figures 6I–K). These data suggest that MYOD1-mediated IGF2 signaling is another effective way for miR-M2-5p to promote cell proliferation.

**Figure 7:**
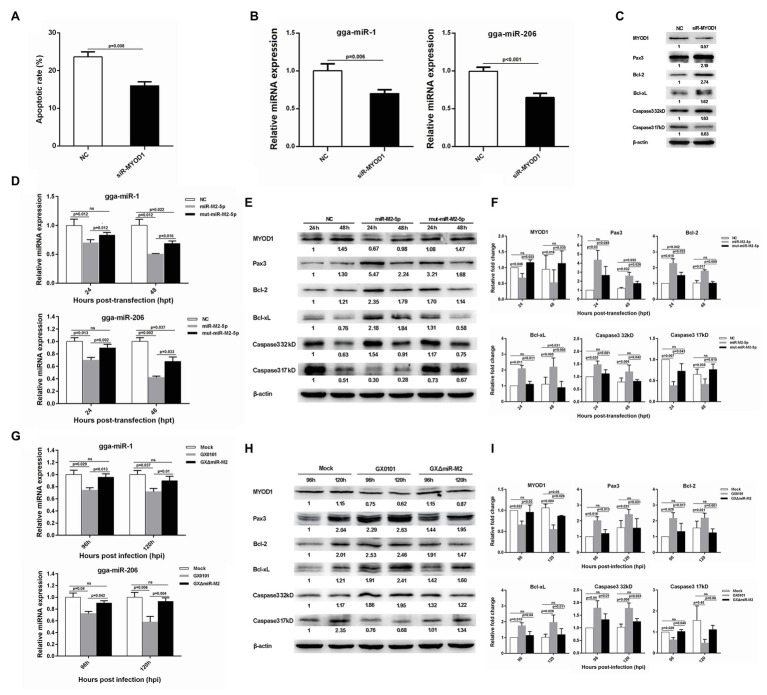
Cell apoptosis and gene expressions involved in miR-M2-5p-mediated MYOD1/Caspase-3 signaling pathway. **(A)** Apoptosis analysis of the MYOD1-silenced CEF cells. CEF cells transfected with MYOD1 siRNA were harvested at 48 hpt for Annexin V-FITC and propidium iodide (PI) staining for apoptosis analysis using flow cytometry. Data are shown as mean ± SD of three independent experiments. **(B)** RT-qPCR analysis of gga-miR-1 and gga-miR-206 expressions in the MYOD1-silenced CEF cells at 48 hpt. **(C)** Western blot analysis of proteins associated with the MYOD1/Caspase3 signaling pathway in the MYOD1-silenced CEF cells at 48 hpt. **(D)** Expressions of gga-miR-1 and gga-miR-206 in CEFs overexpressing miR-M2-5p. **(E)** Expressions of proteins associated with the MYOD1/Caspase3 signaling pathway in CEFs overexpressing miR-M2-5p. **(F)** Relative signal intensities in western blot analysis of the proteins associated with the MYOD1/Caspase3 signaling pathway in CEFs overexpressing miR-M2-5p. **(G)** Expressions of gga-miR-1 and gga-miR-206 in GaHV-2-infected CEFs. **(H)** Expressions of proteins associated with the MYOD1/Caspase3 signaling pathway in GaHV-2-infected CEFs. **(I)** Relative signal intensities in western blot analysis of the proteins associated with the MYOD1/Caspase3 signaling pathway in GaHV-2-infected CEFs. Numbers were normalized to the corresponding signal from the β-actin bands. Error bars are derived from three independent replicates. Values of *p* indicated on columns were used for statistical analyses; ns, no significance.

Interestingly, we also found that siRNA-induced MYOD1 silencing significantly decreased cell apoptosis (Figure 7A). It has been previously reported that MYOD1 regulates the Caspase-3 apoptosis signaling pathway through miRNA-mediated downregulation of Pax3 (Hirai et al., 2010; Zhao et al., 2017). In the present study, we have analyzed major factors involved in this pathway and found that the expressions of gga-miR-1, gga-miR-206, and active Caspase-3 derived from the cleavage of 32 kDa Caspase-3 were downregulated while the expressions of Pax3, Bcl-2, and Bcl-xL were upregulated in MYOD1-silenced cells (Figures 7B,C). To confirm the involvement of miR-M2-5p in MYOD1-mediated apoptosis pathway, major factors in the Caspase-3 apoptotic pathway were further examined in miR-M2-5p-overexpressed CEF cells. As demonstrated in Figures 7D–F, the expression levels of MYOD1, gga-miR-1, gga-miR-206, and active Caspase-3 were similarly downregulated while the expressions of Pax3, Bcl-2, and Bcl-xL were upregulated in CEF cells overexpressing miR-M2-5p, although some of them are not completely recovered to mock levels in the mut-miR-M2-5p transfected cells. The cell apoptosis factors were further detected to be similarly down or upregulated in GX0101-infected CEF cells expressing viral miR-M2-5p while in the mutant GXΔmiR-M2-infected cells they were all rescued (Figures 7G–I), implying that miR-M2-5p inhibits cell apoptosis by suppressing the MYOD1-mediated Caspase-3 signaling pathway.

### Interfering miR-M2-5p Expression in MSB-1 Cells Inhibits Cell Proliferation and Promotes Apoptosis

**Figure 8:**
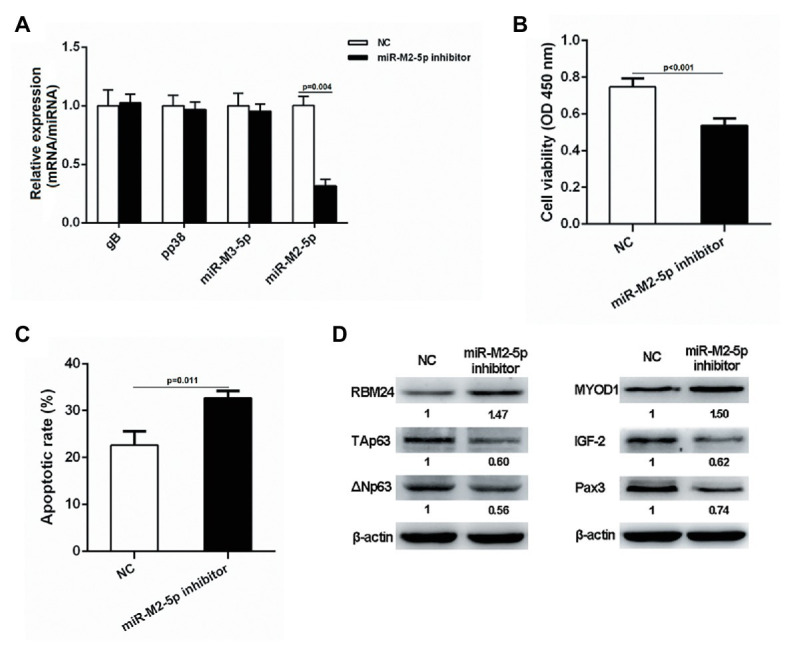
Proliferation and apoptosis of miR-M2-5p inhibitor-transfected MSB-1 cells. **(A)** RT-qPCR analysis of the relative expression levels of viral protein-coding or miRNA genes in MSB-1 cells at 24 hpt with miR-M2-5p inhibitor. **(B,C)** Cell viability and apoptosis of the MSB-1 cells transfected with miR-M2-5p inhibitor. MSB-1 cells transfected with miR-M2-5p inhibitor were harvested at 24 hpt for Annexin V-FITC and propidium iodide (PI) staining for apoptosis analysis using flow cytometry. CCK-8 assay was used to assess cell viability. **(D)** Western blot analysis of proteins associated with the cell proliferation or apoptosis in MSB-1 cells at 24 hpt. Numbers below the blots indicate relative band intensity normalized to β-actin. Error bars are derived from three independent replicates. Values of *p* indicated on columns were used for statistical analyses; ns, no significance.

Since lymphocytes are the main target for the transformation by GaHV-2, we selected the transformed lymphoma cell line MSB-1 to further verify the potential role of miR-M2-5p in MD lymphomagenesis. MSB-1 cells were transfected with miR-M2-5p inhibitor and corresponding negative control for 24 h. As demonstrated in Figure 8A, the RT-qPCR analysis showed that the expression level of miR-M2-5p in the inhibitor-transfected group was significantly lower than that in the negative control group while the relative expression levels of selected viral protein-coding genes, such as *gB* and *pp38* or miRNA miR-M3-5p, are not disturbed. Then, we determined the effects of miR-M2-5p on MSB-1 cell viability and apoptosis. Compared to NC-transfected cells, CCK-8 assay showed that the cell viability of MSB-1 cells was significantly decreased post-transfection with miR-M2-5p inhibitor (Figure 8B). Meanwhile, interfering miR-M2-5p expression remarkably promoted the cisplatin-induced apoptosis in MSB-1 cells (Figure 8C). The major factors involved in regulation of cell proliferation or apoptosis were further detected in MSB-1 cells. Compared to the NC transfected cells, the expression levels of RBM24 and MYOD1 were both upregulated while the expressions of p63, IGF2 and Pax3 were all downregulated in MSB-1 cells transfected with miR-M2-5p inhibitor (Figure 8D). These data are precisely contrary to those in miR-M2-5p overexpressed CEFs, which further support the crucial role of miR-M2-5p in the virally-induced MD lymphomagenesis.

## Discussion

In recent years, viral miRNAs have been increasingly recognized as major determinants for most oncogenic viruses to induce transformation. For the avian oncogenic GaHV-2, early studies have demonstrated that most of the Meq-clustered miRNAs, especially the miR-M4-5p, a virus-encoded miR-155 analog, play critical roles in MD lymphomagenesis (Zhao et al., 2009; Muylkens et al., 2010; Zhao et al., 2011; Yu et al., 2014; Chi et al., 2015). Interestingly, the other viral miRNAs encoded in the Meq-cluster were also preliminarily reported to be implicated in GaHV-2 oncogenesis (Xu et al., 2011; Teng et al., 2015). In the present study, multiple putative mRNA targets for miR-M2-5p were first obtained from a cDNA library. The subsequent experiments, such as DLRA, primarily demonstrated the interactions between miR-M2-5p and the 3'UTRs of six genes, including *RBM24*, *MYOD1, RAC1*, *KIF14*, *WWOX*, and *EMP2*. However in miR-M2-5p overexpressed CEFs, only the expression of genes *RBM24*, *MYOD1*, and *RAC1* was observed to be downregulated. The similar downregulated expressions of *RBM24* and *MYOD1*, but not *RAC1*, were further confirmed in GaHV-2-infected CEFs, which possibly be induced by the viral encoded miRNA. Combined with the data obtained from miRNA-overexpressing and virus-infected cells, we finally identified the host *RBM24* and *MYOD1* genes as two biological targets for miR-M2-5p, which have been frequently reported as important tumor suppressors in previous studies (Kohsaka et al., 2014; Sood et al., 2015; Hua et al., 2016).

RNA-binding protein 24 is a crucial member of RNA-binding proteins (RBPs), which play key roles in post-transcriptional regulation, including mRNA stabilization and translation (Kiledjian et al., 1995; Collier et al., 1998; Poon et al., 2012). In mammalian cancers, many oncogenes and tumor suppressors are under the control of RBPs (Wurth, 2012), among which the RBM24 can bind to the AU/U-rich elements in its target mRNAs and regulate the stability of p21 and p63 mRNA transcripts (Jiang et al., 2014; Xu et al., 2014). Our data in present study revealed that the downregulation of RBM24 mediated by miR-M2-5p actually results in the p63 overexpression and promotes the proliferation of CEF during GaHV-2 infection. As p63 is commonly regarded as an important oncogene (Hibi et al., 2000; Choi et al., 2002; van Bokhoven and Brunner, 2002; Kurita et al., 2004; Hong et al., 2007), the modulation of p63 by miR-M2-5p through targeting RBM24 possibly is one of the potential ways for GaHV-2 to trigger MD lymphomagenesis.

**Figure 9:**
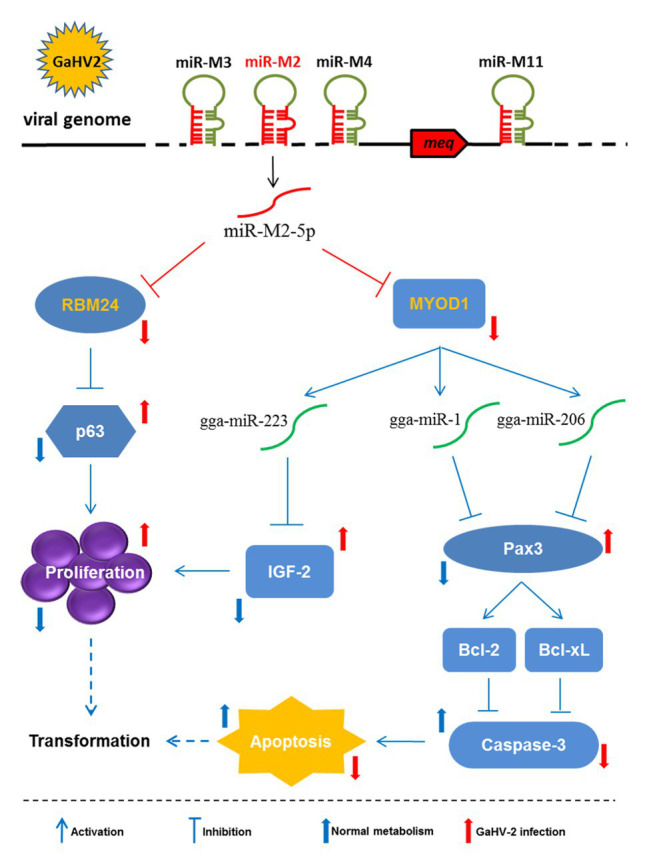
Schematic for the regulatory network mediated by miR-M2-5p in promoting GaHV-2-induced tumorigenesis. The miR-M2-5p triggers the virally-induced T-cell lymphomagenesis by regulating both cell proliferation and apoptosis through multiple smart strategies, including regulating the RBM24-mediated p63 overexpression and the MYOD1-mediated IGF2 and Caspase-3 signaling pathways. In GaHV-2 infection, the expression levels of RBM24 and MYOD1 are both decreased by the viral miR-M2-5p targeting. As one of the consequences, the downregulation of RBM24 mediated by miR-M2-5p actually results in the p63 overexpression and promotes the cell proliferation. On the other hand, the decrease of MYOD1 induces the downregulations of gga-miR-1, gga-miR-206, and gga-miR-223 expressions. The downregulation of gga-miR-223 further inhibits the IGF2 expression and promotes the cell proliferation while the decreases of gga-miR-1 and gga-miR-206 expressions result in the upregulated Pax3 expression and activate two anti-apoptotic factors, Bcl-2, and Bcl-xL, which finally inhibit cell apoptosis. As a final result, all of the target-related signaling pathways involved above may contribute to increase the chances for potential cellular transformation in the virally-induced MD lymphomagenesis.

Myogenic differentiation 1, the other target of miR-M2-5p, is an important transcription factor that regulates cell proliferation, differentiation, and even apoptosis. A direct role for MYOD1 in the regulation of many other genes by binding to their targeted E-box promoter elements containing the sites of “CANNTG,” has been well established previously (Cao et al., 2006; Harford et al., 2017). However, MYOD1 is recently reported to function in upregulating various myogenic miRNAs transcription to inhibit cell proliferation or promote apoptosis (Hirai et al., 2010; Luo et al., 2013; Li et al., 2017; Zhao et al., 2017), demonstrating an indirect role in cancer development. In the present work, our data have confirmed that miR-M2-5p directly targets and inhibits the MYOD1 expression, and thereby induces the downregulated expressions of gga-miR-1, gga-miR-206, and gga-miR-223. On one hand, downregulation of gga-miR-223 induced by the overexpressed miR-M2-5p inhibits the IGF2 expression and promotes the CEF cell proliferation. On the other hand, the miR-M2-5p-induced decrease of gga-miR-1 and gga-miR-206 expressions results in the upregulated Pax3 expression and activates two anti-apoptotic factors, Bcl-2 and Bcl-xL, which finally inhibit CEF cell apoptosis. However, compared to the parental virus-infected cells, the expression of MYOD1 and cell apoptosis were not completely rescued in GXΔmiR-M2-infected CEFs, implying that except for miR-M2-5p the other viral components may be involved in the MYOD1-related signaling that needs to be further studied. Taken together, as demonstrated in Figure 9, our findings propose that as a critical oncomiRNA as we have previously suggested (Teng et al., 2015), miR-M2-5p may trigger the virally-induced MD lymphomagenesis through multiple strategies, including regulating the RBM24-mediated p63 overexpression and the MYOD1-mediated IGF2 and Caspase-3 signaling pathways.

It has been known that most viral miRNAs encoded in the Meq-cluster are potential important regulators contributing to GaHV-2 pathogenicity and oncogenesis (Zhao et al., 2011; Yu et al., 2014; Teng et al., 2015). The most important miR-M4-5p, one of the viral homologs of cellular miR-155 (Zhao et al., 2009), has been characterized as an oncogenic miRNA that recognizes and regulates multiple viral and host mRNA targets (Muylkens et al., 2010; Parnas et al., 2014). Our previous work has revealed that miR-M4-5p activates the expression of c-Myc by targeting LTBP1 and suppressing the TGF-β signaling, thereby playing a critical role in GaHV-2-induced tumors (Chi et al., 2015). Interestingly, miR-M3-5p, another Meq-clustered miRNA, was also demonstrated to contribute to MD oncogenesis by targeting SMAD2, an important component of the TGF-β signaling pathway (Xu et al., 2011). These findings suggest that viral miRNAs may contribute to oncogenesis by co-targeting and regulating the TGF-β signaling pathways. Considering that the miR-M2-5p not only significantly promotes cell proliferation but also protects cells against apoptosis induced by intrinsic apoptotic stimulus, our present work further emphasizes the oncogenic roles of the Meq-clustered miRNAs. In contrast to the Meq-clustered miRNAs that contribute to GaHV-2 pathogenicity and oncogenesis, deletion of the adjacent Mid-cluster miRNAs significantly increased virus pathogenicity and resulted in a 100% mortality in infected birds, implying an inhibitory role of the miRNAs in Mid-cluster in MD lymphomagenesis (Teng et al., 2017). In particular, miR-M11-5p, one of the Mid-cluster miRNAs, has been characterized as a putative tumor suppressor that targets and downregulates the expression of GaHV-2 oncogene *meq* (Teng et al., 2017). Combining the previous reports that miR-M4-5p activates expression of c-Myc (Chi et al., 2015) that has been proposed to form heterodimers with MEQ protein in GaHV-2 infection (Osterrieder et al., 2006), the co-operation of viral and host oncogenes regulated by miRNAs in the Meq‐ and Mid-clusters possibly exerts contrasting roles to keep a balance, which could be an advantage to the virus for establishing, maintaining latency, and/or triggering tumorigenesis. In addition, miR-M7-5p, one of the LAT-clustered miRNAs, has been verified to target the GaHV-2 immediate-early genes *ICP4* and *ICP27*, implying a role contributing to establishment and maintenance of latency (Strassheim et al., 2012). Obviously, the viral miRNAs co-operated with viral and/or host genes in regulating a complicated signaling network in the progress of GaHV-2 pathogenesis and tumorigenesis but more regulatory mechanisms need to be uncovered in the future.

Herein, as an excellent biomodel for the oncogenic herpesvirus-related cancer research, our study on GaHV-2 miR-M2-5p has provided a novel insight in revealing that one single viral miRNA simultaneously regulates both cell proliferation and apoptosis through recognizing and modulating multiple target-related signaling pathways to potentially contribute to herpesvirus pathogenesis and tumorigenesis. It is outstanding that the miR-M2-produced two mature miRNAs, miR-M2-5p and miR-M2-3p, are all stable and highly expressed *in vivo* during the progression of MD disease (Luo et al., 2011). In a recent study, we have tried but failed to identify the mRNA targets for miR-M2-3p using the same approach applied for miR-M2-5p (Supplementary Table S6 and Supplementary Figure 5A). Simultaneously, no significant effect of miR-M2-3p overexpression on CEF cell viability or apoptosis was observed (Supplementary Figures 5B,C). Whether and how miR-M2-3p participates in GaHV-2 oncogenesis needs to be further studied. In addition, due to the overlap of miR-M2 and R-LORF8 gene in GaHV-2 genome, it is difficult to exclude the interference of R-LORF8 when verifying the function of miR-M2-5p using mutant GXΔmiR-M2, although an earlier report has shown that it is not the major determinant of oncogenicity (Zhao et al., 2011). Whether R-LORF8 participates in miR-M2-5p-regulated signaling responsible for cell biological activity requires further study. Along with the development of more powerful mRNA target identification strategies and the applications of CRISPR/Cas9-based gene editing technique in research on herpesvirus biology (Bi et al., 2014; Yuen et al., 2015; Bierle et al., 2016; Zhang et al., 2018, 2019; Luo et al., 2020), further breakthrough in the comprehensive understanding of the biological functions of viral miRNAs can be expected to follow.

## Data Availability Statement

The original contributions presented in the study are included in the article/Supplementary Material, further inquiries can be directed to the corresponding authors.

## Author Contributions

Z-JZ, JL, and G-PZ designed research. Z-JZ and JL analyzed data. Z-JZ, MT, H-ZL, L-PZ, J-LL, and S-JC performed research. Z-JZ, JL, Y-XY, and VN wrote the paper. All authors contributed to the article and approved the submitted version.

### Conflict of Interest

The authors declare that the research was conducted in the absence of any commercial or financial relationships that could be construed as a potential conflict of interest.

## Footnotes

^1^http://www.mirbase.org/cgi-bin/browse.pl

^2^http://www.ncbi.nlm.nih.gov/blast

## References

[ref1] Albanese M. Tagawa T. Buschle A. Hammerschmidt W. (2017). MicroRNAs of Epstein-Barr virus control innate and adaptive antiviral immunity. J. Virol. 91, e01667–e01616. 10.1128/JVI.01667-16, PMID: 28592533 PMC5533892

[ref2] Bartel D. P. (2018). Metazoan microRNAs. Cell 173, 20–51. 10.1016/j.cell.2018.03.006, PMID: 29570994 PMC6091663

[ref3] Bi Y. Sun L. Gao D. Ding C. Li Z. Li Y.. (2014). High-efficiency targeted editing of large viral genomes by RNA-guided nucleases. PLoS Pathog. 10:e1004090. 10.1371/journal.ppat.1004090, PMID: 24788700 PMC4006927

[ref4] Bierle C. J. Anderholm K. M. Wang J. B. Mcvoy M. A. Schleiss M. R. (2016). Targeted mutagenesis of Guinea pig cytomegalovirus using CRISPR/Cas9-mediated gene editing. J. Virol. 90, 6989–6998. 10.1128/JVI.00139-16, PMID: 27226370 PMC4944286

[ref5] Burnside J. Bernberg E. Anderson A. Lu C. Meyers B. C. Green P. J.. (2006). Marek’s disease virus encodes MicroRNAs that map to meq and the latency-associated transcript. J. Virol. 80, 8778–8786. 10.1128/JVI.00831-06, PMID: 16912324 PMC1563840

[ref6] Butz H. Patocs A. (2019). MicroRNAs in endocrine tumors. EJIFCC 30, 146–164. PMID: 31263390 PMC6599198

[ref7] Cao Y. Kumar R. M. Penn B. H. Berkes C. A. Kooperberg C. Boyer L. A.. (2006). Global and gene-specific analyses show distinct roles for Myod and Myog at a common set of promoters. EMBO J. 25, 502–511. 10.1038/sj.emboj.7600958, PMID: 16437161 PMC1383539

[ref8] Chen Y. Fachko D. Ivanov N. S. Skinner C. M. Skalsky R. L. (2019). Epstein-Barr virus microRNAs regulate B cell receptor signal transduction and lytic reactivation. PLoS Pathog. 15:e1007535. 10.1371/journal.ppat.1007535, PMID: 30615681 PMC6336353

[ref9] Chi J. Q. Teng M. Yu Z. H. Xu H. Su J. W. Zhao P.. (2015). Marek’s disease virus-encoded analog of microRNA-155 activates the oncogene c-Myc by targeting LTBP1 and suppressing the TGF-beta signaling pathway. Virology 476, 72–84. 10.1016/j.virol.2014.11.027, PMID: 25528440

[ref10] Choi H. R. Batsakis J. G. Zhan F. Sturgis E. Luna M. A. El-Naggar A. K. (2002). Differential expression of p53 gene family members p63 and p73 in head and neck squamous tumorigenesis. Hum. Pathol. 33, 158–164. 10.1053/hupa.2002.30722, PMID: 11957139

[ref11] Collier B. Goobar-Larsson L. Sokolowski M. Schwartz S. (1998). Translational inhibition in vitro of human papillomavirus type 16 L2 mRNA mediated through interaction with heterogenous ribonucleoprotein K and poly(rC)-binding proteins 1 and 2. J. Biol. Chem. 273, 22648–22656. 10.1074/jbc.273.35.22648, PMID: 9712894

[ref12] Cullen B. R. (2013). MicroRNAs as mediators of viral evasion of the immune system. Nat. Immunol. 14, 205–210. 10.1038/ni.2537, PMID: 23416678 PMC3642974

[ref13] Dang L. Teng M. Li H. Z. Ma S. M. Lu Q. X. Hao H. F.. (2017). Marek’s disease virus type 1 encoded analog of miR-155 promotes proliferation of chicken embryo fibroblast and DF-1 cells by targeting hnRNPAB. Vet. Microbiol. 207, 210–218. 10.1016/j.vetmic.2017.06.015, PMID: 28757026

[ref14] Grey F. (2015). Role of microRNAs in herpesvirus latency and persistence. J. Gen. Virol. 96, 739–751. 10.1099/vir.0.070862-0, PMID: 25406174

[ref15] Guo Y. Li W. Qin J. Lu C. Fan W. (2017). Kaposi’s sarcoma-associated herpesvirus (KSHV)-encoded microRNAs promote matrix metalloproteinases (MMPs) expression and pro-angiogenic cytokine secretion in endothelial cells. J. Med. Virol. 89, 1274–1280. 10.1002/jmv.24773, PMID: 28165144

[ref16] Happel C. Ramalingam D. Ziegelbauer J. M. (2016). Virus-mediated alterations in miRNA factors and degradation of viral miRNAs by MCPIP1. PLoS Biol. 14:e2000998. 10.1371/journal.pbio.2000998, PMID: 27893764 PMC5125562

[ref17] Harford T. J. Kliment G. Shukla G. C. Weyman C. M. (2017). The muscle regulatory transcription factor MyoD participates with p53 to directly increase the expression of the pro-apoptotic Bcl2 family member PUMA. Apoptosis 22, 1532–1542. 10.1007/s10495-017-1423-x, PMID: 28918507 PMC5693709

[ref18] Hibi K. Trink B. Patturajan M. Westra W. H. Caballero O. L. Hill D. E.. (2000). AIS is an oncogene amplified in squamous cell carcinoma. Proc. Natl. Acad. Sci. U. S. A. 97, 5462–5467. 10.1073/pnas.97.10.5462, PMID: 10805802 PMC25851

[ref19] Hirai H. Verma M. Watanabe S. Tastad C. Asakura Y. Asakura A. (2010). MyoD regulates apoptosis of myoblasts through microRNA-mediated down-regulation of Pax3. J. Cell Biol. 191, 347–365. 10.1083/jcb.201006025, PMID: 20956382 PMC2958479

[ref20] Hong S. M. Cho H. Moskaluk C. A. Yu E. Zaika A. I. (2007). p63 and p73 expression in extrahepatic bile duct carcinoma and their clinical significance. J. Mol. Histol. 38, 167–175. 10.1007/s10735-007-9084-7, PMID: 17385050

[ref21] Hua W. F. Zhong Q. Xia T. L. Chen Q. Zhang M. Y. Zhou A. J.. (2016). RBM24 suppresses cancer progression by upregulating miR-25 to target MALAT1 in nasopharyngeal carcinoma. Cell Death Dis. 7:e2352. 10.1038/cddis.2016.252, PMID: 27584791 PMC5059856

[ref22] Huang Y. Qi Y. Ruan Q. Ma Y. He R. Ji Y.. (2011). A rapid method to screen putative mRNA targets of any known microRNA. Virol. J. 8:8. 10.1186/1743-422X-8-8, PMID: 21219658 PMC3025964

[ref23] Jiang Y. Zhang M. Qian Y. Xu E. Zhang J. Chen X. (2014). Rbm24, an RNA-binding protein and a target of p53, regulates p21 expression via mRNA stability. J. Biol. Chem. 289, 3164–3175. 10.1074/jbc.M113.524413, PMID: 24356969 PMC3916521

[ref24] Kiledjian M. Wang X. Liebhaber S. A. (1995). Identification of two KH domain proteins in the alpha-globin mRNP stability complex. EMBO J. 14, 4357–4364. PMID: 7556077 10.1002/j.1460-2075.1995.tb00110.x PMC394520

[ref25] Kincaid R. P. Sullivan C. S. (2012). Virus-encoded microRNAs: an overview and a look to the future. PLoS Pathog. 8:e1003018. 10.1371/journal.ppat.1003018, PMID: 23308061 PMC3534370

[ref26] Kohsaka S. Shukla N. Ameur N. Ito T. Ng C. K. Wang L.. (2014). A recurrent neomorphic mutation in MYOD1 defines a clinically aggressive subset of embryonal rhabdomyosarcoma associated with PI3K-AKT pathway mutations. Nat. Genet. 46, 595–600. 10.1038/ng.2969, PMID: 24793135 PMC4231202

[ref27] Krecic A. M. Swanson M. S. (1999). hnRNP complexes: composition, structure, and function. Curr. Opin. Cell Biol. 11, 363–371. 10.1016/S0955-0674(99)80051-9, PMID: 10395553

[ref28] Kurita T. Mills A. A. Cunha G. R. (2004). Roles of p63 in the diethylstilbestrol-induced cervicovaginal adenosis. Development 131, 1639–1649. 10.1242/dev.01038, PMID: 14998922

[ref29] Li W. Jia X. Shen C. Zhang M. Xu J. Shang Y.. (2016). A KSHV microRNA enhances viral latency and induces angiogenesis by targeting GRK2 to activate the CXCR2/AKT pathway. Oncotarget 7, 32286–32305. 10.18632/oncotarget.8591, PMID: 27058419 PMC5078013

[ref30] Li G. Luo W. Abdalla B. A. Ouyang H. Yu J. Hu F.. (2017). miRNA-223 upregulated by MYOD inhibits myoblast proliferation by repressing IGF2 and facilitates myoblast differentiation by inhibiting ZEB1. Cell Death Dis. 8:e3094. 10.1038/cddis.2017.479, PMID: 28981085 PMC5682648

[ref31] Liu Y. Sun R. Lin X. Liang D. Deng Q. Lan K. (2012). Kaposi’s sarcoma-associated herpesvirus-encoded microRNA miR-K12-11 attenuates transforming growth factor beta signaling through suppression of SMAD5. J. Virol. 86, 1372–1381. 10.1128/JVI.06245-11, PMID: 22013049 PMC3264391

[ref32] Lu Y. Qin Z. Wang J. Zheng X. Lu J. Zhang X.. (2017). Epstein-Barr virus miR-BART6-3p inhibits the RIG-I pathway. J. Innate Immun. 9, 574–586. 10.1159/000479749, PMID: 28877527

[ref33] Luo W. Nie Q. Zhang X. (2013). MicroRNAs involved in skeletal muscle differentiation. J. Genet. Genomics 40, 107–116. 10.1016/j.jgg.2013.02.002, PMID: 23522383

[ref34] Luo J. Sun A. J. Teng M. Zhou H. Cui Z. Z. Qu L. H.. (2011). Expression profiles of microRNAs encoded by the oncogenic Marek’s disease virus reveal two distinct expression patterns in vivo during different phases of disease. J. Gen. Virol. 92, 608–620. 10.1099/vir.0.024158-0, PMID: 21148277

[ref35] Luo J. Teng M. Fan J. Wang F. Zhou L. Deng R.. (2010). Marek’s disease virus-encoded microRNAs: genomics, expression and function. Sci. China Life Sci. 53, 1174–1180. 10.1007/s11427-010-4073-6, PMID: 20953939

[ref36] Luo J. Teng M. Zai X. Tang N. Zhang Y. Mandviwala A.. (2020). Efficient mutagenesis of Marek’s disease virus-encoded microRNAs using a CRISPR/Cas9-based gene editing system. Viruses 12:466. 10.3390/v12040466, PMID: 32325942 PMC7232411

[ref37] Mei M. Zhang M. (2019). Non-coding RNAs in natural killer/T-cell lymphoma. Front. Oncol. 9:515. 10.3389/fonc.2019.00515, PMID: 31263681 PMC6584837

[ref38] Morgan R. Anderson A. Bernberg E. Kamboj S. Huang E. Lagasse G.. (2008). Sequence conservation and differential expression of Marek’s disease virus microRNAs. J. Virol. 82, 12213–12220. 10.1128/JVI.01722-08, PMID: 18842708 PMC2593341

[ref39] Muylkens B. Coupeau D. Dambrine G. Trapp S. Rasschaert D. (2010). Marek’s disease virus microRNA designated Mdv1-pre-miR-M4 targets both cellular and viral genes. Arch. Virol. 155, 1823–1837. 10.1007/s00705-010-0777-y, PMID: 20680360

[ref40] Nagy O. Barath S. Ujfalusi A. (2019). The role of microRNAs in congenital heart disease. EJIFCC 30, 165–178. PMID: 31263391 PMC6599193

[ref41] Osterrieder N. Kamil J. P. Schumacher D. Tischer B. K. Trapp S. (2006). Marek’s disease virus: from miasma to model. Nat. Rev. Microbiol. 4, 283–294. 10.1038/nrmicro1382, PMID: 16541136

[ref42] Parnas O. Corcoran D. L. Cullen B. R. (2014). Analysis of the mRNA targetome of microRNAs expressed by Marek’s disease virus. mBio 5, e01060–e01013. 10.1128/mBio.01060-13, PMID: 24449754 PMC3903288

[ref43] Piedade D. Azevedo-Pereira J. M. (2016). The role of microRNAs in the pathogenesis of herpesvirus infection. Viruses 8:156. 10.3390/v8060156, PMID: 27271654 PMC4926176

[ref44] Poon K. L. Tan K. T. Wei Y. Y. Ng C. P. Colman A. Korzh V.. (2012). RNA-binding protein RBM24 is required for sarcomere assembly and heart contractility. Cardiovasc. Res. 94, 418–427. 10.1093/cvr/cvs095, PMID: 22345307

[ref45] Qin J. Li W. Gao S. J. Lu C. (2017). KSHV microRNAs: tricks of the devil. Trends Microbiol. 25, 648–661. 10.1016/j.tim.2017.02.002, PMID: 28259385 PMC6904892

[ref46] Rehmsmeier M. Steffen P. Hochsmann M. Giegerich R. (2004). Fast and effective prediction of microRNA/target duplexes. RNA 10, 1507–1517. 10.1261/rna.5248604, PMID: 15383676 PMC1370637

[ref47] Ross N. Gandhi M. K. Nourse J. P. (2013). The Epstein-Barr virus microRNA BART11-5p targets the early B-cell transcription factor EBF1. Am. J. Blood Res. 3, 210–224. PMID: 23997984 PMC3755520

[ref48] Sood S. Patel F. D. Ghosh S. Arora A. Dhaliwal L. K. Srinivasan R. (2015). Epigenetic alteration by DNA methylation of ESR1, MYOD1 and hTERT gene promoters is useful for prediction of response in patients of locally advanced invasive cervical carcinoma treated by chemoradiation. Clin. Oncol. (R. Coll. Radiol.) 27, 720–727. 10.1016/j.clon.2015.08.001, PMID: 26344356

[ref49] Strassheim S. Stik G. Rasschaert D. Laurent S. (2012). mdv1-miR-M7-5p, located in the newly identified first intron of the latency-associated transcript of Marek’s disease virus, targets the immediate-early genes ICP4 and ICP27. J. Gen. Virol. 93, 1731–1742. 10.1099/vir.0.043109-0, PMID: 22513387

[ref50] Teng M. Yu Z. H. Sun A. J. Min Y. J. Chi J. Q. Zhao P.. (2015). The significance of the individual Meq-clustered miRNAs of Marek’s disease virus in oncogenesis. J. Gen. Virol. 96, 637–649. 10.1099/jgv.0.000013, PMID: 25502647

[ref51] Teng M. Yu Z. H. Zhao P. Zhuang G. Q. Wu Z. X. Dang L.. (2017). Putative roles as oncogene or tumour suppressor of the mid-clustered microRNAs in Gallid alphaherpesvirus 2 (GaHV2) induced Marek’s disease lymphomagenesis. J. Gen. Virol. 98, 1097–1112. 10.1099/jgv.0.000786, PMID: 28510513 PMC5656797

[ref52] van Bokhoven H. Brunner H. G. (2002). Splitting p63. Am. J. Hum. Genet. 71, 1–13. 10.1086/341450, PMID: 12037717 PMC384966

[ref53] Vojtechova Z. Tachezy R. (2018). The role of miRNAs in virus-mediated oncogenesis. Int. J. Mol. Sci. 19:1217. 10.3390/ijms19041217, PMID: 29673190 PMC5979478

[ref54] Wurth L. (2012). Versatility of RNA-binding proteins in cancer. Comp. Funct. Genomics 2012:178525. 10.1155/2012/178525, PMID: 22666083 PMC3359819

[ref55] Xu S. Xue C. Li J. Bi Y. Cao Y. (2011). Marek’s disease virus type 1 microRNA miR-M3 suppresses cisplatin-induced apoptosis by targeting Smad2 of the transforming growth factor beta signal pathway. J. Virol. 85, 276–285. 10.1128/JVI.01392-10, PMID: 20962090 PMC3014179

[ref56] Xu E. Zhang J. Zhang M. Jiang Y. Cho S. J. Chen X. (2014). RNA-binding protein RBM24 regulates p63 expression via mRNA stability. Mol. Cancer Res. 12, 359–369. 10.1158/1541-7786.MCR-13-0526, PMID: 24375645 PMC3962715

[ref57] Yao Y. Nair V. (2014). Role of virus-encoded microRNAs in avian viral diseases. Viruses 6, 1379–1394. 10.3390/v6031379, PMID: 24662606 PMC3970156

[ref58] Yao Y. Zhao Y. Xu H. Smith L. P. Lawrie C. H. Watson M.. (2008). MicroRNA profile of Marek’s disease virus-transformed T-cell line MSB-1: predominance of virus-encoded microRNAs. J. Virol. 82, 4007–4015. 10.1128/JVI.02659-07, PMID: 18256158 PMC2293013

[ref59] Yu Z. H. Teng M. Sun A. J. Yu L. L. Hu B. Qu L. H.. (2014). Virus-encoded miR-155 ortholog is an important potential regulator but not essential for the development of lymphomas induced by very virulent Marek’s disease virus. Virology 448, 55–64. 10.1016/j.virol.2013.09.017, PMID: 24314636

[ref60] Yuen K. S. Chan C. P. Wong N. H. Ho C. H. Ho T. H. Lei T.. (2015). CRISPR/Cas9-mediated genome editing of Epstein-Barr virus in human cells. J. Gen. Virol. 96, 626–636. 10.1099/jgv.0.000012, PMID: 25502645

[ref61] Zhang Y. Tang N. Luo J. Teng M. Moffat K. Shen Z.. (2019). Marek’s disease virus-encoded microRNA 155 ortholog critical for the induction of lymphomas is not essential for the proliferation of transformed cell lines. J. Virol. 93, e00713–e00719. 10.1128/JVI.00713-19, PMID: 31189706 PMC6694823

[ref62] Zhang Y. Tang N. Sadigh Y. Baigent S. Shen Z. Nair V.. (2018). Application of CRISPR/Cas9 gene editing system on MDV-1 genome for the study of gene function. Viruses 10:279. 10.3390/v10060279, PMID: 29794970 PMC6024840

[ref63] Zhao P. Li X. J. Teng M. Dang L. Yu Z. H. Chi J. Q.. (2015). In vivo expression patterns of microRNAs of *Gallid herpesvirus* 2 (GaHV-2) during the virus life cycle and development of Marek’s disease lymphomas. Virus Genes 50, 245–252. 10.1007/s11262-015-1167-z, PMID: 25666057 PMC4381040

[ref64] Zhao L. Liu Y. Tong D. Qin Y. Yang J. Xue M.. (2017). MeCP2 promotes gastric cancer progression through regulating FOXF1/Wnt5a/beta-catenin and MYOD1/Caspase-3 signaling pathways. EBioMedicine 16, 87–100. 10.1016/j.ebiom.2017.01.021, PMID: 28131747 PMC5474507

[ref65] Zhao Y. Xu H. Yao Y. Smith L. P. Kgosana L. Green J.. (2011). Critical role of the virus-encoded microRNA-155 ortholog in the induction of Marek’s disease lymphomas. PLoS Pathog. 7:e1001305. 10.1371/journal.ppat.1001305, PMID: 21383974 PMC3044692

[ref66] Zhao Y. Yao Y. Xu H. Lambeth L. Smith L. P. Kgosana L.. (2009). A functional MicroRNA-155 ortholog encoded by the oncogenic Marek’s disease virus. J. Virol. 83, 489–492. 10.1128/JVI.01166-08, PMID: 18945769 PMC2612317

[ref67] Zhuang G. Sun A. Teng M. Luo J. (2017). A tiny RNA that packs a big punch: the critical role of a viral miR-155 ortholog in lymphomagenesis in Marek’s disease. Front. Microbiol. 8:1169. 10.3389/fmicb.2017.01169, PMID: 28694799 PMC5483433

