# Supplementary material for: Marek’s Disease Virus (Gallid alphaherpesvirus 2)-Encoded miR-M2-5p Simultaneously Promotes Cell Proliferation and Suppresses Apoptosis Through RBM24 and MYOD1-Mediated Signaling Pathways
Source: Front Microbiol. 2020 Nov 3;11:596422. doi: 10.3389/fmicb.2020.596422 (PMC7669912; doi:10.3389/fmicb.2020.596422)
Supplement: Supplementary file 1 [file Data_Sheet_1.pdf]

# *Supplementary Material*

## Supplementary Figures and Tables

## 1.1 Supplementary Tables

### Table S1. Sequences of RNA oligos.

| Name | Sequence (5´–3´) |
|---|---|
| RBM24 siRNA-383 sense | CCACCUACACCAAGAUCUUTT |
| RBM24 siRNA-383 antisense | AAGAUCUUGGUGUAGGUGGTT |
| RBM24 siRNA-857 sense | CCUACGAGCAAUACCCGUATT |
| RBM24 siRNA-857 antisense | UACGGGUAUUGCUCGUAGGTT |
| RBM24 siRNA-1017 sense | GCAGACAGAUCGUAUGCAATT |
| RBM24 siRNA-1017 antisense | UUGCAUACGAUCUGUCUGCTT |
| MYOD1 siRNA-212 sense | GCCGAUGACUUCUAUGACGTT |
| MYOD1 siRNA-212 antisense | CGUCAUAGAAGUCAUCGGCTT |
| MYOD1 siRNA-628 sense | GCAGGAGGAUGCAUACUACTT |
| MYOD1 siRNA-628 antisense | GUAGUAUGCAUCCUCCUGCTT |
| MYOD1 siRNA-759 sense | GCUACGACAGCAGCUACUATT |
| MYOD1 siRNA-759 antisense | UAGUAGCUGCUGUCGUAGCTT |
| negative siRNA / mimic sense | UUCUCCGAACGUGUCACGUTT |
| negative siRNA / mimic antisense | ACGUGACACGUUCGGAGAATT |
| miR-M2-5p mimic sense | GUUGUAUUCUGCCCGGUAGUCCG |
| miR-M2-5p mimic antisense | GACUACCGGGCAGAAUACAACUU |
| mut-miR-M2-5p mimic sense | G**GU**U**GC**UUCUGCCCGGUAGUCCG |
| mut-miR-M2-5p mimic antisense | GACUACCGGGCAGAAUACAACUU |
| miR-M2-5p inhibitor | CGGACUACCGGGCAGAAUACAAC |
| inhibitor negative control | CAGUACUUUUGUGUAGUACAA |

**Table S2. Primers used for 3′-RACE PCR.**

| Name | Sequence (5′–3′) |
|---|---|
| miR-M2-5p hybrid-primer | CGGRCTRCCGGGCRGRATACAA |
| 3′-RACE out primer | TACCGTCGTTCCACTAGTGATTT |
| 3′-RACE inner primer | CGCGGATCCTCCACTAGTGATTTCACTATAGG |

**Note:** “R” in the sequence of miR-M2-5p hybrid-primer represents for “A” or “G

**Table S3. Primers and oligonucleotides used for making wild or mutated types of psiCHECK-2-3′UTR plasmids.**

| Name | Sequence (5′–3′) | Location | Length |
|---|---|---|---|
| RBM24-3'UTR-F | CCGCTCGAGCATCCAAATCTTGTTGTTAG | 1202-1319 | 118 |
| RBM24-3'UTR-R | GAATGCGGCCGCTGCTACTCCTGCCACTCT | | |
| MYOD1-3'UTR-F | CCGCTCGAGCAATCCAATCTACCAAGTG | 1030-1400 | 371 |
| MYOD1-3'UTR-R | GAATGCGGCCGCCAGGGGTAATAAGGAGAAG | | |
| RAC1-3'UTR-F | CCGCTCGAGGTTATTTCCAGCCCAGTA | 1797-2134 | 338 |
| RAC1-3'UTR-R | GAATGCGGCCGCTTTAGTGGTCGAAAGGGT | | |
| ADRBK2-3'UTR-F | CCGCTCGAGAGTGGTTGTTCTGCGTATG | 4828-4937 | 110 |
| ADRBK2-3'UTR-R | GAATGCGGCCGCACACGACCACAACTATTTACT | | |
| APMAP-3'UTR-F | CCGCTCGAGCCTTGGTTTGACTGCTCT | 1423-1762 | 340 |
| APMAP-3'UTR-R | GAATGCGGCCGCATGTTGCTGCCTCTACCA | | |
| WWOX-3'UTR-F | CCGCTCGAGTCATCGTAACTGGTGGGT | 1062-1573 | 512 |
| WWOX-3'UTR-R | GAATGCGGCCGCCCTCCTTGGGTTACACTT | | |
| EMP2-3'UTR-F | CCGCTCGAGTGGCTATTCCTTTGTCTTA | 626-1263 | 638 |
| EMP2-3'UTR-R | GAATGCGGCCGCTTTGCAGAGCAAGTGGTT | | |
| KIF14-3'UTR-F | CCGCTCGAGAAAACTACCGTATGAGACAAA | 6734-7114 | 381 |
| KIF14-3'UTR-R | GAATGCGGCCGCAAGCAACACCGCACCTCA | | |
| GNB1-3'UTR-F | CCGCTCGAGCAGCGGATTGTTCTGTTA | 2737-3052 | 316 |
| GNB1-3'UTR-R | GAATGCGGCCGCTCATGTCAAACTGCTTTATT | | |
| ATP8A2-3'UTR-F | CCGCTCGAGTAATGTCCTCTGGTCGTTT | 9058-9176 | 119 |
| ATP8A2-3'UTR-R | GAATGCGGCCGCGCTGTTCAAAGCTACAAATT | | |
| BACE2-3'UTR-F | CCGCTCGAGAGGAAACAACAAAACGAA | 2925-3482 | 558 |
| BACE2-3'UTR-R | GAATGCGGCCGCTGACGGATTGGGAGATTC | | |
| FGD3-3'UTR-F | CCGCTCGAGCACTGACATTCCGTTCGC | 3856-4385 | 530 |
| FGD3-3'UTR-R | GAATGCGGCCGCCAAATACAAGTGAACAGGAAAA | | |
| PIGG-3'UTR-F | CCGCTCGAGAAAGCTACGTGGAGATACC | 2365-2862 | 498 |
| PIGG-3'UTR-R | GAATGCGGCCGCGTGTAACGCAGGACAAGC | | |
| Mut-RBM24-3'UTR-F | TCGAGGCATCCAAATCTTGTTGTTAGAGGGGAGTGG TTCTCGGCCATGGCTAGTTGC | 1201-1250 | 50 |
| Mut-RBM24-3'UTR-R | GGCCGCAACTAGCCATGGCCGAGAACCACTCCCCT CTAACAACAAGATTTGGATGCC | 1201-1250 | 50 |
| Mut-MYOD1-3'UTR-F | TCGAGGGAGAATAGGGAACCTGGCTCTTGTTTAGCC AGGAGAAAAGAAATCTACAGC | 1311-1360 | 50 |
| Mut-MYOD1-3'UTR-R | GGCCGCTGTAGATTTCTTTTCTCCTGGCTAAACAAG AGCCAGGTTCCCTATTCTCCC | 1311-1360 | 50 |
| Mut-RAC1-3'UTR-F | TCGAGAATGTTCTGTACATCCTCAGCTGGCAGGATA ACTCTGTTGAACTACACCCTGC | 2071-2121 | 51 |
| Mut-RAC1-3'UTR-R | GGCCGCAGGGTGTAGTTCAACAGAGTTATCCTGCCA GCTGAGGATGTACAGAACATTC | 2071-2121 | 51 |
| Mut-KIF14-3'UTR-F | TCGAGTCTTTTTGCACTTTTCGGCTTTGGAGGGCAT AGCTTTCATGCTGAACTAGTGC | 6950-7000 | 51 |

| | | | |
|---|---|---|---|
| Mut-KIF14-3'UTR-R | GGCCGCACTAGTTCAGCATGAAAGCTATGCCCTCCA AAGCCGAAAAGTGCAAAAAGAC | 6950-7000 | 51 |
| Mut-WWOX-3'UTR-F | TCGAGATGTTAAGTACTAATGGGAAATGGGGATAAC CCCGGGTTAAAGGGACAATGGC | 1449-1499 | 51 |
| Mut-WWOX-3'UTR-R | GGCCGCCATTGTCCCTTTAACCCGGGGTTATCCCCAT TTCCCATTAGTACTTAACATC | 1449-1499 | 51 |
| Mut-EMP2-3'UTR-F | TCGAGTGTACTGTGTTCTTAATATGAAACAGATAACT TAACAAAAAACAACCAACAGC | 825-875 | 51 |
| Mut-EMP2-3'UTR-R | GGCCGCTGTTGGTTGTTTTTTGTTAAGTTATCTGTTT CATATTAAGAACACAGTACAC | 825-875 | 51 |

**Table S4. Primers used for qRT-PCR in this study.**

| Name | Sequence (5′–3′) |
|---|---|
| RT-qPCR-RBM24-F | AGGATCATGCAGCCAGGTTT |
| RT-qPCR-RBM24-R | GTGGATAGACATAGTGAGCTGGG |
| RT-qPCR-MYOD1-F | GGCGGCTCAGCAAGGTCAAC |
| RT-qPCR-MYOD1-R | AGGCTCTCGATGTAGCGGATGG |
| RT-qPCR-RAC1-F | AGAAGAAGCTGACTCCTATCACCTACC |
| RT-qPCR-RAC1-R | GCCTCGCTGTGTAAGTGCTGAG |
| RT-qPCR-WWOX-F | TGGTGGTCGTGTCCTCAGAGTC |
| RT-qPCR-WWOX-R | AAGGCGTCGGTTCAGCTCATTG |
| RT-qPCR-EMP2-F | GGTCATGCTGTTCCATCTCCATTCC |
| RT-qPCR-EMP2-R | TAACTTCTTGCCAGTCCTGTTGTGC |
| RT-qPCR-KIF14-F | TTGAGTTGTTCTTAGGAAGCCAGGAAG |
| RT-qPCR-KIF14-R | AAGAGGAGATAGCAGCAGCATTATGAC |
| RT-qPCR-gB-F | TCTAGGGCATGGCACACGAC |
| RT-qPCR-gB-R | GAATACGGAAACACAGGAGCGG |
| RT-qPCR-pp38-F | CCGAAAGACAAAACCCAAAT |
| RT-qPCR-pp38-R | ATGTAACCAGCATATAAGAACGC |
| RT-qPCR-p63-F | TTCGGCAAGGAACTCATGGCATAC |
| RT-qPCR-p63-R | TGGTGCTGCTGTTGCTGCTG |
| RT-qPCR-p21-F | GTGACTGCTGCTGCTGAGGATG |
| RT-qPCR-p21-R | AACTACAGACTCGGCATTGCTTCG |
| miR-M2-5p reverse-transcribed | CTCAACTGGTGTCGTGGAGTCGGCAATTCAGTTGA GCGGACTAC |
| miR-M3-5p reverse-transcribed | CTCAACTGGTGTCGTGGAGTCGGCAATTCAGTTGA GGCGGGAGA |
| miR-neg reverse-transcribed | CTCAACTGGTGTCGTGGAGTCGGCAATTCAGTTGA GAACGTCTC |
| gga-miR-1 reverse-transcribed | CTCAACTGGTGTCGTGGAGTCGGCAATTCAGTTGA GTACATACT |
| gga-miR-206 reverse-transcribed | CTCAACTGGTGTCGTGGAGTCGGCAATTCAGTTGA GCCACACAC |
| gga-miR-223 reverse-transcribed | CTCAACTGGTGTCGTGGAGTCGGCAATTCAGTTGA GGGGGTATT |
| U6 reverse-transcribed | CTCAACTGGTGTCGTGGAGTCGGCAATTCAGTTGA GAAAAACATGG |
| RT-qPCR-miR-M2-5p-F | ACACTCCAGCTGGGGTTGTATTCTGCCCGGT |
| RT-qPCR-miR-M3-5p-F | ACACTCCAGCTGGGATGAAAATGTGAAACCTC |
| RT-qPCR-miR-neg-F | ACACTCCAGCTGGGGTACTGCGCGTGGA |
| RT-qPCR-gga-miR-1-F | ACACTCCAGCTGGGTGGAATGTAAAGAAG |
| RT-qPCR-gga-miR-206-F | ACACTCCAGCTGGGTGGAATGTAAGGAAGT |
| RT-qPCR-gga-miR-223-F | ACACTCCAGCTGGGTGTCAGTTTGTCAAA |
| RT-qPCR-miR-universal-R | CTCAACTGGTGTCGTGGAGTCGGCAATTCAG |

| | |
|---|---|
| RT-qPCR-GAPDH-F | GGTAGTGAAGGCTGCTGCTGATG |
| RT-qPCR-GAPDH-R | AGTCCACAACACGGTTGCTGTATC |
| U6-F | CTCGCTTCGGCAGCACA |
| U6-R | AACGCTTCACGAATTTGCGT |

**Table S5. Putative host mRNA targets for miR-M2-5p acquired by hybrid-PCR**

<table>
<tr><th>No.</th><th>Gene Abb.</th><th>Acc. No.</th><th>mRNA : miRNA interaction</th><th>Location</th></tr>
<tr><td>1</td><td>RAC1</td><td>XM_015294138.1</td><td>5’ UCCUCAGCUGGCAGGAAUACAG3’<br>|   | |||||  |||||||||<br>3’ GCCUGAUGGCCCGUCUUAUGUUG5’</td><td>3'UTR</td></tr>
<tr><td>2</td><td>ARIH1</td><td>NM_001030387.1</td><td>5’ GGGGCCCUGGGACAGAACGCGG3’<br> ||||  | || ||||| ||||<br>3’ GCCUGAUGGCCCGUCUUAUGUUG5’</td><td>3'UTR</td></tr>
<tr><td>3</td><td>RBM24</td><td>NM_001012863.2</td><td>5’ UUAGAGGGGAGUGGUAGUGCGG3’<br>|  |  |   |  | |||||||<br>3’ GCCUGAUGGCCCGUCUUAUGUUG5’</td><td>3'UTR</td></tr>
<tr><td>4</td><td>ADRBK2</td><td>XM_015275493.1</td><td>5’ GGGGCUGUUGUGCAGUGUACAG3’<br> ||||||||| |||| ||||||<br>3’ GCCUGAUGGCCCGUCUUAUGUUG5’</td><td>3'UTR</td></tr>
<tr><td>5</td><td>APMAP</td><td>NM_001006177.1</td><td>5’ UAUUAAUGGCUGGCGAAUACGG3’<br>|          |  ||||||||<br>3’ GCCUGAUGGCCCGUCUUAUGUUG5’</td><td>3'UTR</td></tr>
<tr><td>6</td><td>WWOX</td><td>NM_001030574.2</td><td>5’ UAAUGGGAAAUGGGGAAUACGG3’<br>|     |    | |||||||||<br>3’ GCCUGAUGGCCCGUCUUAUGUUG5’</td><td>3'UTR</td></tr>
<tr><td>7</td><td>VMA21</td><td>NM_001031127.1</td><td>5’ UCCUUUGUGAAGCAGAACACAA3’<br>|   ||||   |||||| ||||<br>3’ GCCUGAUGGCCCGUCUUAUGUUG5’</td><td>3'UTR</td></tr>
<tr><td>8</td><td>EMP2</td><td>XM_414935.5</td><td>5’ CUUAAUAUGAAACAGAAUACAA3’<br>|  | |||    ||||||||||<br>3’ GCCUGAUGGCCCGUCUUAUGUUG5’</td><td>3'UTR</td></tr>
<tr><td>9</td><td>MYOD1</td><td>NM_204214.2</td><td>5’ GGAACCUGGCUCUUGAAUACGG3’<br> | ||       | ||||||||<br>3’ GCCUGAUGGCCCGUCUUAUGUUG5’</td><td>3'UTR</td></tr>
<tr><td>10</td><td>TPT1</td><td>NM_205398.1</td><td>5’ GAAAUGUUAACAAAUCAAACAU3’<br>   ||  |     |  | |||<br>3’ GCCUGAUGGCCCGUCUUAUGUUG5’</td><td>3'UTR</td></tr>
<tr><td>11</td><td>PIGG</td><td>XM_015280488.1</td><td>5’ CAAUUUGUUGUGCAGAGUGCGA3’<br>|   |||||| |||||||||||<br>3’ GCCUGAUGGCCCGUCUUAUGUUG5’</td><td>3'UTR</td></tr>
<tr><td>12</td><td>SET</td><td>NM_001030691.2</td><td>5’ AAAUACCUUGAGCAGAACACAG3’<br>       ||| |||||| ||||<br>3’ GCCUGAUGGCCCGUCUUAUGUUG5’</td><td>3'UTR</td></tr>
<tr><td>13</td><td>ENC1</td><td>XM_015277651.1</td><td>5’ GGCUUCCCGGUCAGAAAUGGAA3’<br> |  |  | |   | |||| ||<br>3’ GCCUGAUGGCCCGUCUUAUGUUG5’</td><td>3'UTR</td></tr>
<tr><td>14</td><td>SIRT7</td><td>NM_001291971.1</td><td>5’ UGCGACGGCUGGUGAGUGCGGG3’<br>|| |  | | |||| |    ||<br>3’ GCCUGAUGGCCCGUCUUAUGUUG5’</td><td>3'UTR</td></tr>
<tr><td>15</td><td>TBL1XR1</td><td>NM_001031369.1</td><td>5’ GGGACGUAAUUCUGCCAAACUU3’<br> ||||       ||  | ||<br>3’ GCCUGAUGGCCCGUCUUAUGUUG5’</td><td>3'UTR</td></tr>
<tr><td>16</td><td>KIF14</td><td>XM_422190.5</td><td>5’ UCGGCUUUGGAGGGCAAUGCAA3’<br>| |||| | | | | |||||||<br>3’ GCCUGAUGGCCCGUCUUAUGUUG5’</td><td>3'UTR</td></tr>
<tr><td>17</td><td>GPR64</td><td>XM_015272778.1</td><td>5’ UAUGUCUGAGACGGAAUACGAC3’<br>|  ||    |   | |    |<br>3’ GCCUGAUGGCCCGUCUUAUGUUG5’</td><td>3'UTR</td></tr>
</table>

<table>
<tr><td>18</td><td>RGMB</td><td>XM_424860.5</td><td>5’ AGGGCCCCAGCUCUGACUGCAG3’<br> ||||  | |  | || |||||<br>3’ GCCUGAUGGCCCGUCUUAUGUUG5’</td><td>3'UTR</td></tr>
<tr><td>19</td><td>WNT5A</td><td>XM_015292956.1</td><td>5’ CUGGCUAUCUUUCGAAACACAG3’<br>| |||||||   || || ||||<br>3’ GCCUGAUGGCCCGUCUUAUGUUG5’</td><td>3'UTR</td></tr>
<tr><td>20</td><td>GNB1</td><td>NM_001012835.2</td><td>5’ ACUUCAAGAAAGCUGGAUGCAA3’<br>    | |    || ||||||||<br>3’ GCCUGAUGGCCCGUCUUAUGUUG5’</td><td>3'UTR</td></tr>
<tr><td>21</td><td>ATP8A2</td><td>XM_004938793.2</td><td>5’ UUCCCUCCCAGACUGAAUACAA3’<br>|   || || | | ||||||||<br>3’ GCCUGAUGGCCCGUCUUAUGUUG5’</td><td>3'UTR</td></tr>
<tr><td>22</td><td>BACE2</td><td>XM_015301191.1</td><td>5’ UGGACUGACUAUCUGAAUACAA3’<br>||||||| |   | ||||||||<br>3’ GCCUGAUGGCCCGUCUUAUGUUG5’</td><td>3'UTR</td></tr>
<tr><td>23</td><td>TDO2</td><td>XM_420377.5</td><td>5’ CUGUAAUCUGCUAAGGGUAUGG3’<br>| |    |||   |||||||||<br>3’ GCCUGAUGGCCCGUCUUAUGUUG5’</td><td>3'UTR</td></tr>
<tr><td>24</td><td>CTSO</td><td>NM_001031129.1</td><td>5’ CCAUACCCUUAGCAGAUUACAG3’<br>|       |  ||||| |||||<br>3’ GCCUGAUGGCCCGUCUUAUGUUG5’</td><td>3'UTR</td></tr>
<tr><td>25</td><td>FGD3</td><td>XM_414331.5</td><td>5’ UAUACUGCCAACUGGGAUACGA3’<br>|  ||||||   ||||||||||<br>3’ GCCUGAUGGCCCGUCUUAUGUUG5’</td><td>3'UTR</td></tr>
<tr><td>26</td><td>SMAD5</td><td>XM_015293968.1</td><td>5’ AAGCGUCCAAGAAAGAAUACAG3’<br>  |  | |  |  |||||||||<br>3’ GCCUGAUGGCCCGUCUUAUGUUG5’</td><td>5'UTR</td></tr>
<tr><td>27</td><td>UPF2</td><td>XM_015297566.1</td><td>5’ CAGACUUGCCUGGGUAACACAA3’<br>| ||||  |  | | || ||||<br>3’ GCCUGAUGGCCCGUCUUAUGUUG5’</td><td>5'UTR</td></tr>
<tr><td>28</td><td>EIF5B</td><td>NM_001038496.1</td><td>5’ AACAAGAGCGAGGACAGUGCAA3’<br>   |  | || | | |||||||<br>3’ GCCUGAUGGCCCGUCUUAUGUUG5’</td><td>CDS</td></tr>
<tr><td>29</td><td>GTF3A</td><td>NM_001113571.1</td><td>5’ AUGUGGUUGUGACAGAAUGCAA3’<br>  |    |  | ||||||||||<br>3’ GCCUGAUGGCCCGUCUUAUGUUG5’</td><td>CDS</td></tr>
<tr><td>30</td><td>ATP5C1</td><td>NM_001278096.1</td><td>5’ ACCACCAGUGAGCAGAGUGCAA3’<br>   || | || |||||||||||<br>3’ GCCUGAUGGCCCGUCUUAUGUUG5’</td><td>CDS</td></tr>
<tr><td>31</td><td>EIF5B</td><td>NM_001038496.1</td><td>5’ AACAAGAGCGAGGACAGUGCAA3’<br>   |  | || | | |||||||<br>3’ GCCUGAUGGCCCGUCUUAUGUUG5’</td><td>CDS</td></tr>
<tr><td>32</td><td>ANXA11</td><td>NM_001012903.3</td><td>5’ UGGACAUACGUGCAGAAUACAA3’<br>|||||   || |||||||||||<br>3’ GCCUGAUGGCCCGUCUUAUGUUG5’</td><td>CDS</td></tr>
<tr><td>33</td><td>ANXA4</td><td>XM_015297501.1</td><td>5’ GAGACUGCUCAGGAGACUACAG3’<br>  |||||||  | ||| |||||<br>3’ GCCUGAUGGCCCGUCUUAUGUUG5’</td><td>CDS</td></tr>
<tr><td>34</td><td>RPL21</td><td>XM_001233392.3</td><td>5’ AGGACUAACGGGAAGGAUCCGG3’<br> |||||| |||| ||||| |||<br>3’ GCCUGAUGGCCCGUCUUAUGUUG5’</td><td>CDS</td></tr>
<tr><td>35</td><td>LYAR</td><td>XM_004936167.2</td><td>5’ GACAGCACUUUGCAGAAUCAGG3’<br>   |  |||  |||||||  ||<br>3’ GCCUGAUGGCCCGUCUUAUGUUG5’</td><td>CDS</td></tr>
<tr><td>36</td><td>ATP5I</td><td>NM_001097534.2</td><td>5’ GAGGAGCUCGAACGGAUUGCAA3’<br>  ||   |||  ||||||||||<br>3’ GCCUGAUGGCCCGUCUUAUGUUG5’</td><td>CDS</td></tr>
</table>

<table>
<tr><td>37</td><td>ENAH</td><td>NM_204300.1</td><td>5' ACCCAGUGCCAAUGGAGUACAG3'<br>        |   ||||||||||<br>3' GCCUGAUGGCCCGUCUUAUGUUG5'</td><td>CDS</td></tr>
<tr><td>38</td><td>HNRNPD</td><td>NM_001031143.1</td><td>5' ACAACAACUACUACGGAUACAG3'<br>   || |||     ||||||||<br>3' GCCUGAUGGCCCGUCUUAUGUUG5'</td><td>CDS</td></tr>
<tr><td>39</td><td>RPS6</td><td>NM_205225.2</td><td>5' AAACGCCAGGAGCAGAUUGCGA3'<br>         | ||||| |||||<br>3' GCCUGAUGGCCCGUCUUAUGUUG5'</td><td>CDS</td></tr>
<tr><td>40</td><td>RTF1</td><td>XM_421139.5</td><td>5' AGACCGGGCUCGACUGGAGCAA3'<br> |  | | |  |   ||  ||||<br>3' GCCUGAUGGCCCGUCUUAUGUUG5'</td><td>CDS</td></tr>
<tr><td>41</td><td>ZNHIT6</td><td>XM_422364.5</td><td>5' GUCUCCUCGGUCAGAUGUACAA3'<br>    |  | |   |  ||||||<br>3' GCCUGAUGGCCCGUCUUAUGUUG5'</td><td>CDS</td></tr>
<tr><td>42</td><td>TOP1</td><td>NM_205110.1</td><td>5' AACACCGGCACAAAGAGCACAA3'<br>   || | |    |||| ||||<br>3' GCCUGAUGGCCCGUCUUAUGUUG5'</td><td>CDS</td></tr>
<tr><td>43</td><td>RPL5</td><td>NM_204581.4</td><td>5' CUCAGGGCCCAGGAGCGUGCAG3'<br>|  |  |||  | || ||||||<br>3' GCCUGAUGGCCCGUCUUAUGUUG5'</td><td>CDS</td></tr>
<tr><td>44</td><td>PSMC5</td><td>NM_001292069.1</td><td>5' GGCUGUUGCCAAGGUGAUGCAG3'<br> |   |  |    | |||||||<br>3' GCCUGAUGGCCCGUCUUAUGUUG5'</td><td>CDS</td></tr>
<tr><td>45</td><td>RPL30</td><td>XM_015282954.1</td><td>5' GUAAAUACGUGCUAGGAUACAA3'<br>   | |||  | ||||||||||<br>3' GCCUGAUGGCCCGUCUUAUGUUG5'</td><td>CDS</td></tr>
<tr><td>46</td><td>TRIO</td><td>XM_015282226.1</td><td>5' AUUCUUGUUUAAAAACAGUAUC3'<br>    |||||    |  |<br>3' GCCUGAUGGCCCGUCUUAUGUUG5'</td><td>CDS</td></tr>
<tr><td>47</td><td>CHMP5</td><td>NM_001277399.1</td><td>5' GAGGGGCCUGCAAAGAAUACAG3'<br>   |   |||   |||||||||<br>3' GCCUGAUGGCCCGUCUUAUGUUG5'</td><td>CDS</td></tr>
<tr><td>48</td><td>CMSS1</td><td>NM_001285926.1</td><td>5' UCUGCUUGCAGCACAUGAAUAU3'<br>|  |||  | |     | |||<br>3' GCCUGAUGGCCCGUCUUAUGUUG5'</td><td>CDS</td></tr>
<tr><td>49</td><td>GNAS</td><td>XM_015296547.1</td><td>5' UGACUGUCGGGACAUUAUUCAG3'<br>||  |  | || ||  || |||<br>3' GCCUGAUGGCCCGUCUUAUGUUG5'</td><td>CDS</td></tr>
<tr><td>50</td><td>PIGX</td><td>XM_015277133.1</td><td>5' ACCACCUCUGUCAGUGGUACAG3'<br>   ||  |||   | |||||||<br>3' GCCUGAUGGCCCGUCUUAUGUUG5'</td><td>CDS</td></tr>
<tr><td>51</td><td>ITSN1</td><td>XM_015299993.1</td><td>5' AGCUGGAACGGGCAGAGCAAGA3'<br> |    | ||||||||| ||||<br>3' GCCUGAUGGCCCGUCUUAUGUUG5'</td><td>CDS</td></tr>
</table>

**Table S6. Putative host mRNA targets for miR-M2-3p acquired by hybrid-PCR**

<table>
<tr><th>No.</th><th>Gene Abb.</th><th>Acc. No.</th><th>mRNA : miRNA interaction</th><th>Location</th></tr>
<tr><td>1</td><td>CCL3</td><td>XM_015296023.1</td><td>5’ ACAATTAGATGCAGCAGTCC 3’<br>|    |     ||| |||||||<br>3’ UUCGAUAAGACGCCGUCAGGC5’</td><td>3'UTR</td></tr>
<tr><td>2</td><td>PSMA2</td><td>NM_001012860.2</td><td>5’ GGGTGCAAATGCAGCAGTCC3’<br>||||      ||| |||||||<br>3’ UUCGAUAAGACGCCGUCAGGC5’</td><td>3'UTR</td></tr>
<tr><td>3</td><td>RNF14</td><td>NM_001030623.2</td><td>5’ CTGTGCCTTGCTGCCAGTCC3’<br>||    || ||| ||||||<br>3’ UUCGAUAAGACGCCGUCAGGC5’</td><td>3'UTR</td></tr>
<tr><td>4</td><td>SIPA1L1</td><td>XM_010711501.2</td><td>5’ TGCATCTCTGCGTGCACTCC3’<br> |  | | |     ||| |||<br>3’ UUCGAUAAGACGCCGUCAGGC5’</td><td>3'UTR</td></tr>
<tr><td>5</td><td>NFκBIZ</td><td>NM_001006254.1</td><td>5’ CACTGTCAGTCAGGCAGTCC3’<br>| |     |  ||||||||<br>3’ UUCGAUAAGACGCCGUCAGGC5’</td><td>3'UTR</td></tr>
<tr><td>6</td><td>MAPK6</td><td>NM_001030549.1</td><td>5’ AGCTTATTCTGAGGCTATCC3’<br>|| |||||||| |||  |||<br>3’ UUCGAUAAGACGCCGUCAGGC5’</td><td>3'UTR</td></tr>
<tr><td>7</td><td>ARID4A</td><td>XM_015287509.1</td><td>5’ GTTGGAAACTTTGGCAGTCC3’<br>|    |  || |||||||||<br>3’ UUCGAUAAGACGCCGUCAGGC5’</td><td>3'UTR</td></tr>
<tr><td>8</td><td>RFC1</td><td>XM_015285652.1</td><td>5’ TGAGAAAAATGCTGCAGACC3’<br>|   |   ||| |||| ||<br>3’ UUCGAUAAGACGCCGUCAGGC5’</td><td>3'UTR</td></tr>
<tr><td>9</td><td>CNEP1R1</td><td>NM_001198857.2</td><td>5’ AGCAATTCTTCCGCCCGTCC3’<br>||    | || || | ||||<br>3’ UUCGAUAAGACGCCGUCAGGC5’</td><td>3'UTR</td></tr>
<tr><td>10</td><td>CLIC4</td><td>XM_015297737.1</td><td>5’ CTATTCCTGCTCGGTTGTCC3’<br>||  |    |||| ||||<br>3’ UUCGAUAAGACGCCGUCAGGC5’</td><td>3'UTR</td></tr>
<tr><td>11</td><td>ARIH1</td><td>NM_001030387.1</td><td>5’ GGGACAGAACGCGGAAGTCA3’<br>|||  |     |||| ||||<br>3’ UUCGAUAAGACGCCGUCAGGC5’</td><td>3'UTR</td></tr>
<tr><td>12</td><td>AKR1E2</td><td>NM_001243590.1</td><td>5’ CTTTCTTAATGCGGCTGTCC3’<br>|  |  |||||| ||||<br>3’ UUCGAUAAGACGCCGUCAGGC5’</td><td>3'UTR</td></tr>
<tr><td>13</td><td>RDH12</td><td>XM_421193.5</td><td>5’ CTATCCATGTACATCAGTCC3’<br>|   | | |  ||||||<br>3’ UUCGAUAAGACGCCGUCAGGC5’</td><td>3'UTR</td></tr>
<tr><td>14</td><td>ZNF385B</td><td>XM_013093934.1</td><td>5’ AGCATCTTTATCTGCAATCC3’<br>||  | |||  | ||| |||<br>3’ UUCGAUAAGACGCCGUCAGGC5’</td><td>3'UTR</td></tr>
<tr><td>15</td><td>ACADSB</td><td>NM_001031240.1</td><td>5’ GGTCTCTTCATGTGCAGTCC3’<br>|| || |||     |||||||<br>3’ UUCGAUAAGACGCCGUCAGGC5’</td><td>3'UTR</td></tr>
<tr><td>16</td><td>KCTD10</td><td>XM_415191.5</td><td>5’ ACTTTGAGTGAGGGCAGTCC3’<br>|  |||  |    ||||||||<br>3’ UUCGAUAAGACGCCGUCAGGC5’</td><td>3'UTR</td></tr>
<tr><td>17</td><td>PTBP3</td><td>XM_004949517.2</td><td>5’ TTATCTGTTCACGGAAGTCC3’<br>|   ||  ||| |||||<br>3’ UUCGAUAAGACGCCGUCAGGC5’</td><td>3'UTR</td></tr>
<tr><td>18</td><td>ZNHIT3</td><td>XM_015296154.1</td><td>5’ AAGCCATTCTGCAGCACCCC3’<br>|||| ||||||| |||   ||<br>3’ UUCGAUAAGACGCCGUCAGGC5’</td><td>3'UTR</td></tr>
</table>

<table>
<tr><td>19</td><td>ROCK2</td><td>XM_015276087.1</td><td>5’ TGCGTCATCATGGGCAGTCC3’<br>|  |  |||  ||||||||<br>3’ UUCGAUAAGACGCCGUCAGGC5’</td><td>3'UTR</td></tr>
<tr><td>20</td><td>RCAN1</td><td>NM_001278079.1</td><td>5’ GTTCTGCTTCCTGGCAGTCC3’<br>|  ||| ||  |||||||||<br>3’ UUCGAUAAGACGCCGUCAGGC5’</td><td>3'UTR</td></tr>
<tr><td>21</td><td>ABCB10</td><td>XM_015284390.1</td><td>5’ TGCTGATTTTGTGGCAGTCC3’<br>| | ||||||||||||||<br>3’ UUCGAUAAGACGCCGUCAGGC5’</td><td>3'UTR</td></tr>
<tr><td>22</td><td>GUCY1A2</td><td>XM_015852224.1</td><td>5’ GCTAGGAGCAGACCAATTCT3’<br>|    |  | |    |||||<br>3’ UUCGAUAAGACGCCGUCAGGC5’</td><td>3'UTR</td></tr>
<tr><td>23</td><td>PSMD1</td><td>NM_001012600.3</td><td>5’ CTGTTCAGTGCATGCAGCCC3’<br>| |   |    |||| ||<br>3’ UUCGAUAAGACGCCGUCAGGC5’</td><td>3'UTR</td></tr>
<tr><td>24</td><td>CRNKL1</td><td>XM_419315.5</td><td>5’ AAGTTCTATCTGTGCAATCC3’<br>||||| | |    ||| |||<br>3’ UUCGAUAAGACGCCGUCAGGC5’</td><td>3'UTR</td></tr>
<tr><td>25</td><td>C5H11ORF58</td><td>XM_015286530.1</td><td>5’ ACACGATGAAGCTGCGGTCC3’<br>|  | ||   || |||||||<br>3’ UUCGAUAAGACGCCGUCAGGC5’</td><td>CDS</td></tr>
<tr><td>26</td><td>PRPF6</td><td>XM_417426.5</td><td>5’ AGCCTGTTCTCAAGCGGTCC3’<br>|| |||||||   |||||||<br>3’ UUCGAUAAGACGCCGUCAGGC5’</td><td>CDS</td></tr>
<tr><td>27</td><td>ZC3H11A</td><td>NM_001031030.1</td><td>5’ ACAAGCTTTCTGTGCAGTCC3’<br>|     |||    |||||||<br>3’ UUCGAUAAGACGCCGUCAGGC5’</td><td>CDS</td></tr>
<tr><td>28</td><td>MPP4</td><td>XM_015289463.1</td><td>5’ TCAGCTGTTGTCTGCAGTCC3’<br>||  | |||||||<br>3’ UUCGAUAAGACGCCGUCAGGC5’</td><td>CDS</td></tr>
<tr><td>29</td><td>ATF4</td><td>NM_204880.2</td><td>5’ CCTGTGGCTCTGTGCGGTCC3’<br>|   |    |||||||<br>3’ UUCGAUAAGACGCCGUCAGGC5’</td><td>CDS</td></tr>
<tr><td>30</td><td>HNRNPA3</td><td>XM_015289584.1</td><td>5’ ACAGTGGACAGCAGCAGTCC3’<br>|   ||  | || |||||||<br>3’ UUCGAUAAGACGCCGUCAGGC5’</td><td>CDS</td></tr>
<tr><td>31</td><td>FAM18B1</td><td>NM_001030699.1</td><td>5’ AGCAGTTTTTGCGGCAGACC3’<br>||    |||||||||||  ||<br>3’ UUCGAUAAGACGCCGUCAGGC5’</td><td>CDS</td></tr>
<tr><td>32</td><td>PRKG1</td><td>XM_015278708.1</td><td>5’ GAACTCCTGACTGGCAGTCC3’<br>|| ||  |    |||||||||<br>3’ UUCGAUAAGACGCCGUCAGGC5’</td><td>CDS</td></tr>
<tr><td>33</td><td>ZC3H11A</td><td>NM_001031030.1</td><td>5’ ACAAGCTTTCTGTGCAGTCC3’<br>|     |||    |||||||<br>3’ UUCGAUAAGACGCCGUCAGGC5’</td><td>CDS</td></tr>
<tr><td>34</td><td>MRPS2</td><td>XM_015279792.1</td><td>5’ CTTCTATCCTGCGACCCTCC3’<br>    |||| ||||| |  |||<br>3’ UUCGAUAAGACGCCGUCAGGC5’</td><td>CDS</td></tr>
<tr><td>35</td><td>PHF3</td><td>XM_015284854.1</td><td>5’ CTGTAATTCCTCGGCGCTCC3’<br>|| ||||  ||||| |||<br>3’ UUCGAUAAGACGCCGUCAGGC5’</td><td>CDS</td></tr>
<tr><td>36</td><td>ZFR</td><td>XM_015848318.1</td><td>5’ GGAGGAACTTCAGGCAGTCC3’<br>||   |  ||  ||||||||<br>3’ UUCGAUAAGACGCCGUCAGGC5’</td><td>CDS</td></tr>
<tr><td>37</td><td>RPL27</td><td>XM_015299594.1</td><td>5’ CGGTATTCTGTTGACATTCC3’<br>|||  | |  || || |||<br>3’ UUCGAUAAGACGCCGUCAGGC5’</td><td>CDS</td></tr>
</table>

<table>
<tr><th></th><th></th><th></th><th></th><th></th></tr>
<tr><td>38</td><td>ELF1</td><td>NM_001006269.1</td><td>5’ TAGATGATCCTGAGCAGTCC3’<br>|| || ||    |||||||<br>3’ UUCGAUAAGACGCCGUCAGGC5’</td><td>CDS</td></tr>
<tr><td>39</td><td>CCDC88C</td><td>XM_015287749.1</td><td>5’ TGAAAGAACAGCACCAGTCC3’<br>|   |  | ||  ||||||<br>3’ UUCGAUAAGACGCCGUCAGGC5’</td><td>CDS</td></tr>
<tr><td>40</td><td>HS6ST1</td><td>NM_204482.1</td><td>5’ AGAGGCTTCTTCACCGGTCC3’<br>||    |||| |  ||||||<br>3’ UUCGAUAAGACGCCGUCAGGC5’</td><td>CDS</td></tr>
<tr><td>41</td><td>MDH1</td><td>NM_001006395.2</td><td>5’ GGCAATTCTTACGGCGTTCC3’<br>||    | || ||||| |||<br>3’ UUCGAUAAGACGCCGUCAGGC5’</td><td>CDS</td></tr>
<tr><td>42</td><td>CCDC91</td><td>XM_015292478.1</td><td>5’ AGGCTTTGATGCAGCAGTCA3’<br>||||| |  ||| ||||||<br>3’ UUCGAUAAGACGCCGUCAGGC5’</td><td>CDS</td></tr>
<tr><td>43</td><td>MAP1A</td><td>XM_015292140.1</td><td>5’ AGCAGAGGAAAAGGCAGTCC3’<br> ||   |      ||||||||<br>3’ UUCGAUAAGACGCCGUCAGGC5’</td><td>CDS</td></tr>
<tr><td>44</td><td>AUS6</td><td>XM_015280096.1</td><td>5’ GAAGCTATACGCTGCAGTCC3’<br>||     |  || |||||||<br>3’ UUCGAUAAGACGCCGUCAGGC5’</td><td>CDS</td></tr>
<tr><td>45</td><td>TGFB2</td><td>NM_001031045.3</td><td>5’ CCTCTCCGTGCTGCGTGTCC3’<br>||   |  ||   ||||<br>3’ UUCGAUAAGACGCCGUCAGGC5’</td><td>CDS</td></tr>
</table>

## 1.2 Supplementary Figures

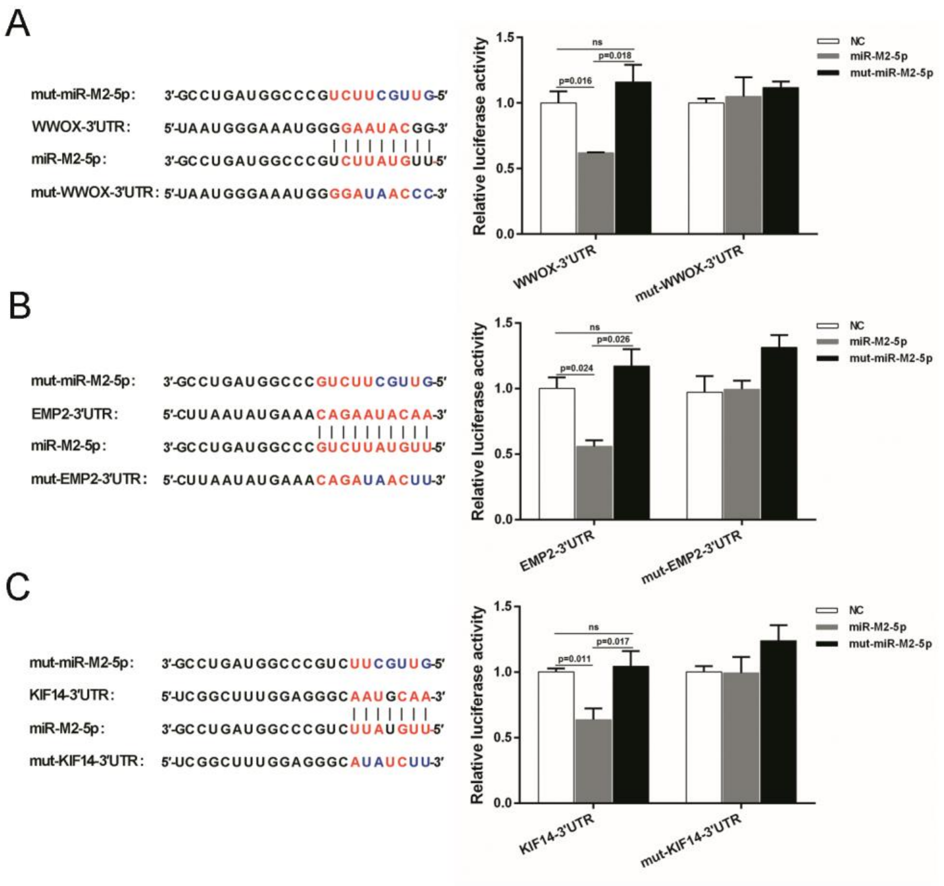


**Supplementary Figure 1.** Interactions between miR-M2-5p and 3′UTRs of candidate mRNA targets determined by dual luciferase reporter assay (DLRA). (A-C) DLRA performed for confirming the interactions between miR-M2-5p and 3′UTRs of mRNA candidate target genes, *WWOX*, *EMP2* and *KIF14*. The colored seed sequence of miRNA or binding sites of 3′UTRs and the corresponding mutants are shown on the left side. The wild type or mutated 3′UTRs of candidate mRNA targets were cloned into the downstream of *Renilla* luciferase in psiCHECK-2 vector, and then were co-transfected into 293T cells with the miR-M2-5p, mut-miR-M2-5p or miRNA negative control (NC) mimics, respectively. *Firefly* and *Renilla* luciferase activities

were measured at 48 h post-transfection using the dual luciferase reporter system (Promega). *Firefly* luciferase was served as the internal control. For each luciferase assay, relative luciferase activity was normalized with respect to miRNA NC. Results are shown as the mean ± SD of three independent experiments. Independent sample *t*-test was used to analyze the statistical differences between groups. P values indicated on columns were used for statistical analyses; ns, no significance.

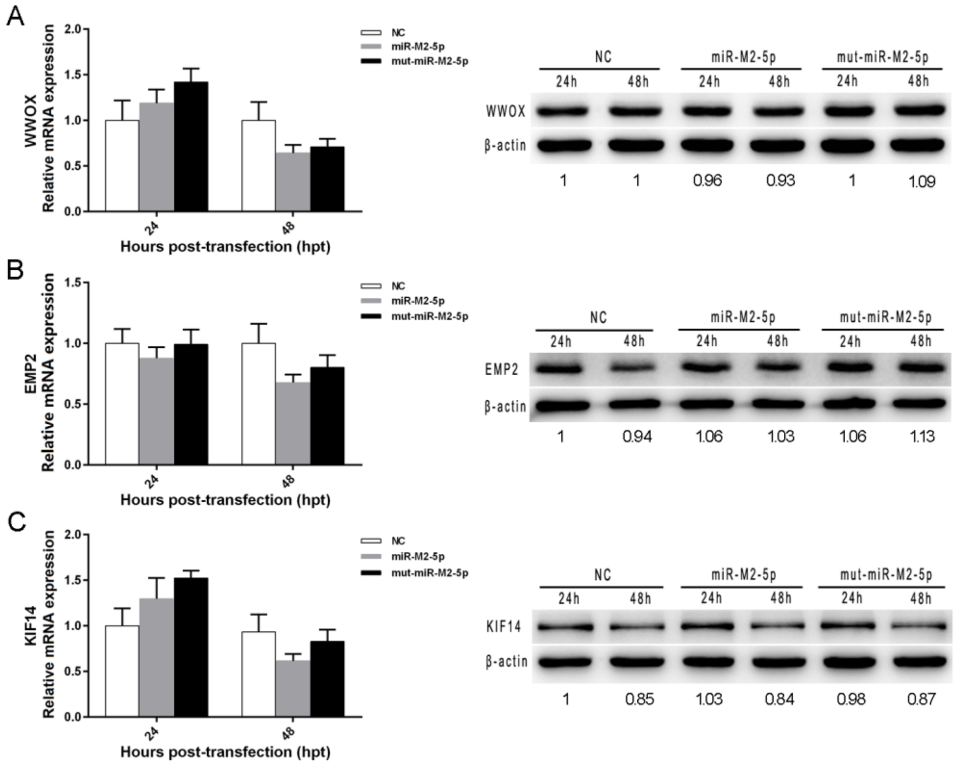


**Supplementary Figure 2.** (A-C) qRT-PCR and western blot analysis performed to analyze the inhibitory effect of miR-M2-5p on candidate targets WWOX, EMP2, and KIF14 respectively. Numbers below the blots indicate relative band intensity normalized to β-actin. Error bars are derived from three independent replicates.

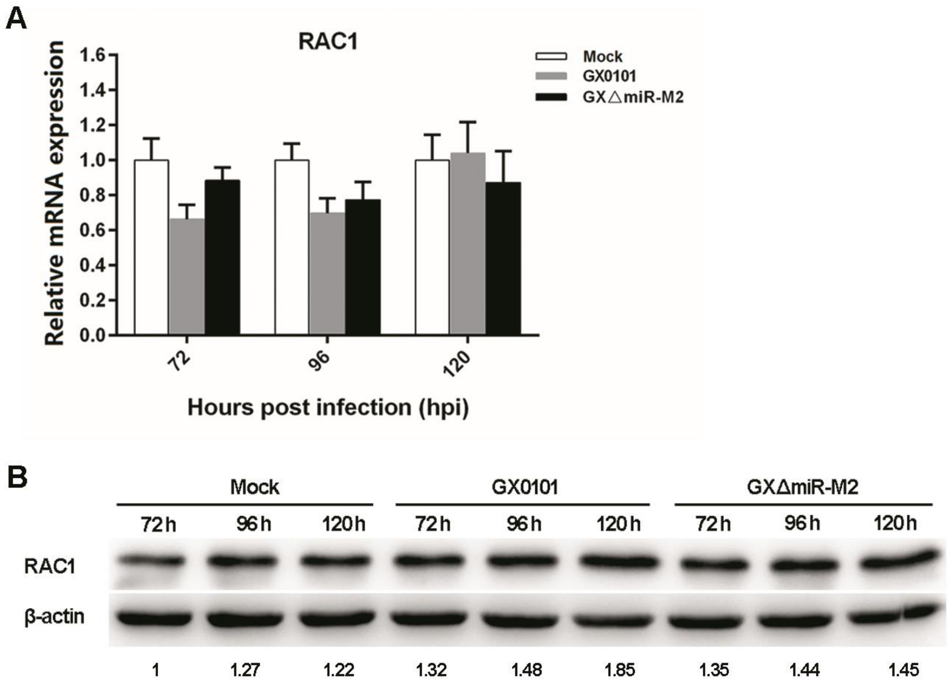


**Supplementary Figure 3.** Expression levels of RAC1 in CEFs infected with GaHV2. (A) Expression levels of the RAC1 mRNA genes in virus-infected CEFs determined by qRT-PCR. Results are shown as the mean ± SD of three independent experiments. Independent sample t-test was used to analyze the statistical differences between groups. (B) Expression levels of the RAC1 proteins in virus-infected CEFs determined by western blot. Numbers below the blots indicate relative band intensity normalized to β-actin.

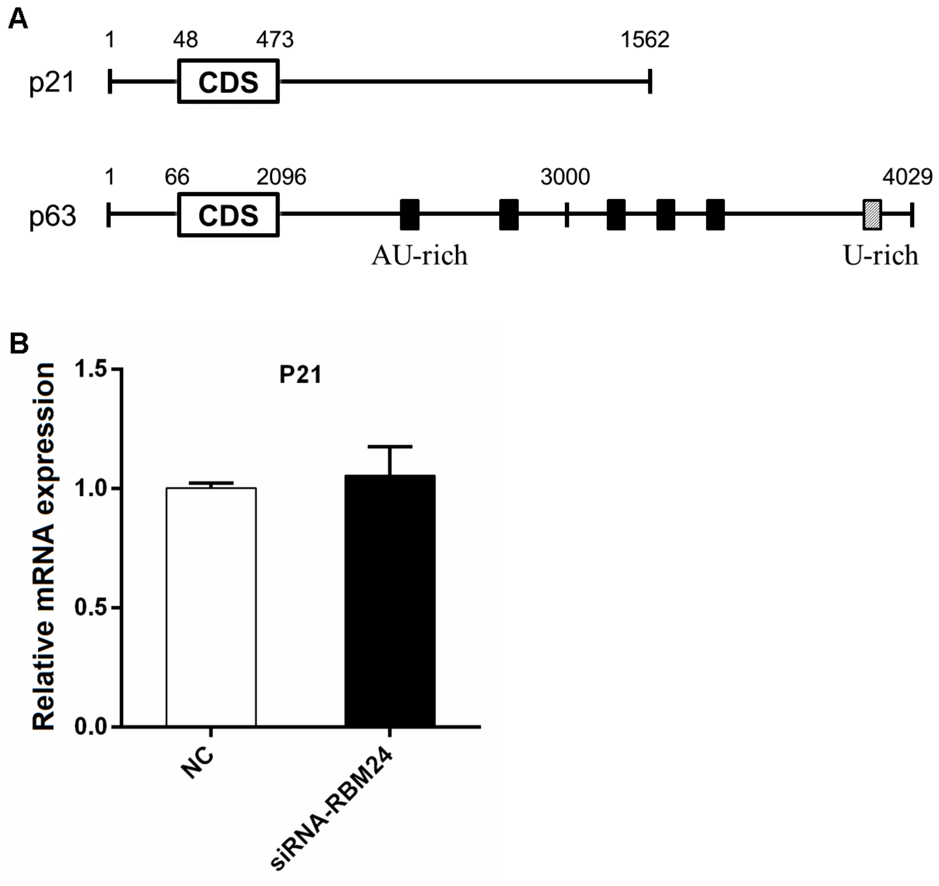


**Supplementary Figure 4.** (A) Schematic representations of chicken *p63* and *p21* transcripts. The AU- or U-rich elements were shown in shaded boxes. The sequence comparison was done using the online tool, BLAST. (B) RT-qPCR analysis of *p21* expressions in the RBM24-silenced CEF cells at 48 hours post-transfection. Error bars are derived from three independent replicates.

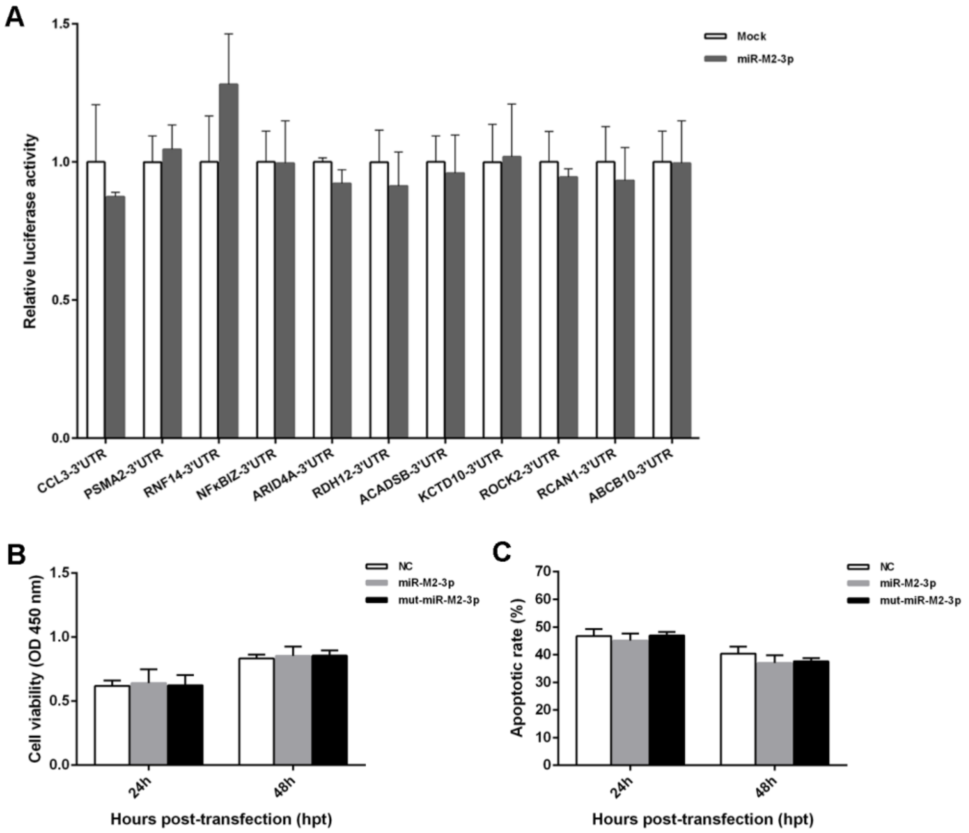


**Supplementary Figure 5.** (A) The first round of DLRA for primary analysis of the interactions between miR-M2-3p and 3'UTRs of eleven mRNA containing perfect base pairing to the seed region of miR-M2-3p, including CCL3, PSMA2, RNF14, NFκBIZ, ARID4A, RDH12, ACADSB, KCTD10, ROCK2, RCAN1, and ABCB10. (B & C) Cell viability and apoptosis of miR-M2-3p-overexpressed CEF cells. At different time points post miRNA transfection, the CEF cells were harvested for Annexin V-FITC and propidium iodide (PI) staining for apoptosis analysis using flow cytometry. CCK-8 assay was used to assess cell viability. Error bars are derived from three independent replicates.
